# Exacerbated salmonellosis in poly(ADP-ribose) polymerase 14-deficient mice

**DOI:** 10.1128/spectrum.02971-25

**Published:** 2025-12-30

**Authors:** Madhukar Vedantham, Lauri Polari, Tiia Rissanen, Arto Tapio Pulliainen

**Affiliations:** 1 Institute of Biomedicine, University of Turku 169300, Turku, Finland; 2 Cell Biology, Biosciences, Faculty of Science and Engineering, Åbo Akademi University, Turku, Finland; 3 InFLAMES Research Flagship Center, Åbo Akademi University 1040, Turku, Finland; 4 Department of Biostatistics, University of Turku 8058, Turku, Finland; University of Guelph College of Biological Science, Guelph, Ontario, Canada

**Keywords:** *Salmonella*, gastroenteritis, infection, inflammation, Parp14

## Abstract

*Salmonella enterica* subspecies *enterica* serovar Typhimurium is an enteropathogen annually causing millions of acute infections ranging from gastroenteritis to life-threatening systemic disease. Strong mucosal inflammation, a process with incomplete molecular understanding, is characteristic of *S*. Typhimurium gastroenteritis. Here, we investigated functions of the nucleocytoplasmic protein poly(ADP-ribose) polymerase (Parp14) in the mouse model of *S*. Typhimurium infection. Using a systemic Parp14 knockout approach, we found that infected Parp14-deficient mice suffered from exacerbated histopathology, in particular, in the large intestine, that is, increased immune cell infiltration, goblet cell loss, and epithelial erosion. A bulk tissue and single-cell RNA-Seq analysis supplemented with TaqMan qPCR assays was executed to obtain molecular-level functional approximations. We found evidence of a defective Th17 response in the infected Parp14-deficient mice. This parallels the known cell-intrinsic regulatory function of Parp14 in Th17 cell differentiation. However, based on immunohistochemistry, we found that Parp14 was also expressed by macrophages and, in particular, by epithelial cells across the mucosal tissues in small intestine, cecum, and large intestine. The bulk tissue and epithelial cell subtype single-cell RNA-Seq data comparison revealed a plausible epithelial cell-specific transcriptomic signature defective in the infected Parp14-deficient mice. Downregulation of *ApoA1*, *Spink1,* and *Sst*, encoding apolipoprotein A1, serine protease inhibitor Kazal-type 1, and somatostatin, respectively, was characteristic of this defective transcriptomic signature. We conclude that Parp14 is an integral part of the physiological response to *S*. Typhimurium infection and that Parp14 acts as a multi-cell-type pleiotropic regulator of mucosal inflammation.

**IMPORTANCE:**

Eukaryotic cells rely on dynamic cell-signaling mechanisms to mount responses to external perturbations, such as an invading bacterial pathogen. The PARP protein family is a group of enzymes catalyzing a protein post-translational modification known as ADP-ribosylation. PARP1, the founding member, has received considerable research interest, in particular in cancer. However, recent data imply that PARP1 and, in particular, the other PARPs have regulatory functions in inflammatory responses. Yet, the mechanistic basis and, more importantly, the physiological relevance have largely remained elusive. Our study with the systemic Parp14-deficient mice provides compelling *in vivo* evidence that Parp14 is an integral part of the physiological response to *S.* Typhimurium infection.

## INTRODUCTION

*Salmonella enterica* subspecies *enterica* serovar Typhimurium (hereafter *S*. Typhimurium) is a food- and waterborne enteropathogen annually causing millions of acute infections ranging from the self-limiting non-invasive gastroenteritis to life-threatening invasive systemic disease (1, 2). The globally emerging antibiotic-resistant strains complicate the clinical management of the most severe forms of *S*. Typhimurium infection (2–4). Based on the analyses of patient tissue samples, *S*. Typhimurium elicits mucosal inflammation, in particular in the terminal ileum and colon, characterized by a massive neutrophil influx (5, 6). It is believed that the expression of bacterial virulence factors (7, 8), such as flagella and type I fimbriae, drives the initial contacts between *S*. Typhimurium and colon epithelial cells, the enterocytes, through the protective barrier created by the colon lumen microbiota (9) and dense epithelial cell mucous layer (10). It appears evident that *S*. Typhimurium also actively interacts with trans-epithelial and/or lamina propria-associated dendritic cells as well as macrophages (7, 8). Upon initial contact with the host cells, *S*. Typhimurium is thought to utilize two different type 3 secretion systems (TTSS-1 and TTSS-2) and a plethora of TTSS effector proteins to drive deeper colon wall invasion and intracellular as well as extracellular replication (7, 8). Some of the TTSS effects are counterintuitively driving mucosal inflammation, which is additionally activated by the classical innate immunity receptors, such as Toll-like receptors recognizing pathogen-associated molecular patterns (7, 8, 11, 12). It is believed that *S*. Typhimurium benefits from colon inflammation to outcompete the colon lumen microbiota (13, 14), that is, via the creation of gut dysbiosis, and to obtain nutrients boosting the colon lumen bacterial replication (15). It remains incompletely understood how and at which point the mucosal inflammation turns anti-bacterial and how the tissue homeostasis is restored in the colon. Elucidation of molecular mechanisms regulating the mucosal inflammation in *S*. Typhimurium infection could be useful for the development of novel host-targeted anti-bacterial pharmaceuticals.

Eukaryotic cells rely on dynamic cell-signaling events to mount responses to external perturbations, such as an invading bacterial pathogen. Post-translational modifications influence the location, half-life, and activity of proteins and thereby serve as a powerful regulatory mechanism of cell signaling. Poly(ADP-ribose) polymerase 14 (Parp14) is a multidomain ADP-ribosyltransferase (ART) enzyme of the Parp protein family (16). Parp14 was identified as a Stat6-interacting protein (17) with a postulated role as a transcriptional co-factor in interleukin-4 (IL-4)-induced Stat6-dependent gene expression (17–20). Parp14 catalyzes protein ADP-ribosylation, including itself (21–23), which refers to the covalent conjugation of an ADP-ribose moiety from nicotinamide adenine dinucleotide (NAD+) onto the substrate protein with simultaneous release of nicotinamide (16). The functional outcomes of Parp14-catalyzed protein ADP-ribosylation have remained largely unknown. Regulation of macrophage activation via ADP-ribosylation of Stat proteins has been proposed (24, 25). The recent work on the identification of Parp14 as the key ART of the interferon-γ (IFN-γ)-inducible protein ADP-ribosylation response is expected to pave the way for a better understanding of the cellular and physiological functions of Parp14 (22, 26–29). Parp14 functions that are independent of its ART activity, such as ADP-ribose-dependent scaffolding and ADP-ribose modification reversal, also appear possible, as exemplified by the recent work on ADP-ribose binding and ADP-ribose hydrolysis activities of the Parp14 macrodomains (30, 31). In respect to bacterial infections, one previous *in vitro* phenotypic study employing Parp14 depletion has been published (32). The Parp14-deficient RAW264.7 macrophages contained more viable *S*. Typhimurium bacteria and, upon *S*. Typhimurium infection, produced less microbicidal nitric oxide as well as had a defective expression pattern of a number of inflammation-related genes, such as *Ifnb1*, *Ccl5*, *Cxcl10*, and *Ifit1* (32). It therefore appears plausible that Parp14 has functional relevance to mount a controlled physiological response to a bacterial infection.

We hypothesized that Parp14 is involved in the regulation of anti-bacterial mucosal inflammation, and, if so, its malfunction could play a role in the development of bacterial gastroenteritis. We explored the expression of Parp14 and the effect of its systemic genetic deficiency in the mouse streptomycin-pretreatment model of *S*. Typhimurium infection (8, 33). Exacerbated salmonellosis was witnessed in Parp14-deficient mice. This phenotype paralleled defective transcriptomic signatures with functional significance in infection and inflammation responses. Given the multi-cell-type expression of Parp14 (epithelial cells, macrophages, T cells, B cells), as found in this and previous studies, for example, references 17–20, 25, 32, 34, Parp14 appears to act as a multi-cell-type pleiotropic regulator of mucosal inflammation.

## MATERIALS AND METHODS

### Mouse experimentation

#### Colony breeding

Our colony of Parp14-deficient mice was established based on the previously described body-wide Parp14 knockout mice (34), kindly provided by Adam Hurlstone (University of Manchester, UK), in a specific-pathogen-free area at the Central Animal Laboratory of the University of Turku, with free access to a soy-free diet and water *ad libitum*. The Parp14 knockout mice were backcrossed for 10 generations to a C57BL/6N background before starting the experiments (35).

#### *Salmonella* infection

Experiments with female C57BL/6N mice aged 6–8 weeks were performed as described (33). The mice were allocated to two groups, that is, phosphate-buffered saline (PBS) control and *Salmonella* infection groups, with similar starting body weights. The naturally streptomycin-resistant SL1344 strain of *S*. Typhimurium was purchased from the Culture Collection University of Gothenburg (CCUG 51871), Gothenburg, Sweden. Bacteria were grown for 12 h at 37°C in Luria-Bertani (LB) medium with shaking, diluted 1:20 in fresh medium, and sub-cultured for 4 h with shaking. Bacteria were washed twice and suspended in ice-cold sterile PBS. Water and food were withdrawn 4 h before *per os* (p.o.) treatment with 20 mg of streptomycin (75 μL of sterile solution or 75 μL of sterile water). Afterward, animals were supplied with water and food *ad libitum*. At 20 h after streptomycin treatment, water and food were withdrawn again for 4 h before the mice were orally gavaged with 10^8^ colony-forming units (CFU) of *Salmonella* (50 μL suspension in PBS, p.o.) or with PBS (50 μL). Thereafter, drinking water *ad libitum* was offered immediately and food 2 h post-infection (p.i.). At the indicated times p.i., mice were sacrificed by CO_2_ asphyxiation, organs were weighed, colon length was measured, and tissue samples were processed for bacterial viability quantitation (CFU/g) (samples collected in cold PBS), histological analyses (samples collected in 4% paraformaldehyde), and RNA analysis (samples collected in liquid nitrogen).

### Quantitation infection severity

#### Enumeration of viable bacteria

Fecal pellets were placed in 500 μL of ice-cold PBS and suspended to homogeneity on ice by vortexing and pipetting. The distal small intestine, cecum, proximal and mid-large intestine, mesenteric lymph nodes, spleens, and livers were removed aseptically and homogenized in ice-cold PBS at +4°C by using stainless steel balls (IKA 5 mm stainless steel balls, Fisher Scientific) and a compact bead mill (TissueLyser LT, Qiagen). CFUs were determined by plating different dilutions on LB agar plates (streptomycin, 50 μg/mL). Plates were incubated at 37°C for approximately 12 h before counting the colonies.

#### Histopathological analysis

Formalin-fixed paraffin-embedded (FFPE) tissues were cut into longitudinal 5 µm-thick sections prior to hematoxylin and eosin (HE) staining. HE staining was performed using standard methods. All samples were scanned using a Pannoramic 1000 Slide scanner (3DHistech, Budapest, Hungary) with a 20× objective and analyzed with a Pannoramic Viewer (3DHistech, software version 1.15.4). Histopathology was scored based on four variables, that is, (i) immune cell infiltration to the gut wall as 0 (healthy/neglectable numbers of immune cells), 1 (minor level of immune cells), 2 (moderate level of immune cells), 3 (high level of immune cells indicative of a severe inflammation); (ii) edema of the gut wall as 0 (healthy/neglectable edema), 1 (minor edema), 2 (moderate edema), 3 (strong edema indicative of a severe inflammation); (iii) epithelial erosion of the gut wall as 0 (healthy/neglectable erosion), 1 (mild loss of epithelial cells), 2 (moderate loss of epithelial cells), 3 (strong loss of epithelial cells and erosion going through muscular lamina, indicative of a severe inflammation); and (iv) loss of goblet cells of the gut wall as 0 (healthy/neglectable loss of goblet cells), 1 (minor loss of goblet cells), 2 (moderate loss of goblet cells), 3 (only a few sporadic goblet cells left, indicative of a severe inflammation). Two people performed the scoring independently and blinded to the animal groups to obtain the average scores for statistical analysis.

#### Statistical analyses

The statistical analysis for studying association of variables (tissue variables, histology scores, bacterial load variables, weights) with mice group (wild-type [wt] vs Parp14-KO) was analyzed separately by termination day. Tissue variables (spleen, liver, colon length) and bacterial load variables (proximal colon, liver, spleen, MLNs, fecal pellets, distal small intestine) were summarized with descriptive statistics, and associations between mice groups were studied using the Kruskal-Wallis test. Histology scores (edema, immune infiltration, goblet cell loss, erosion) were measured in the distal small intestine, proximal colon, and whole cecum. Associations between mice groups and variables were also studied by the Kruskal-Wallis test. Weight percentages were summarized with descriptive statistics. Differences between groups in the 5 day follow-up were analyzed using the Friedman test because the normality assumption was not met. The Wilcoxon signed-rank test was used to study the difference over days. The normality of variables was evaluated visually and tested with the Shapiro-Wilk test. Due to the non-normality of the continuous variables, nonparametric methods were used. All tests were performed as two-tailed, and the statistical significance level was set at 0.05. The analyses were carried out using the SAS system, version 9.4 for Windows (SAS Institute Inc., Cary, NC, USA).

### Parp14 immunohistochemistry

The 5 µm-thick FFPE sections were stained for Parp14 with mouse monoclonal antibody (sc-377150, Santa Cruz Biotechnology, dilution 1:500) and detected using the mouse-specific HRP-DAB (ABC) detection immunohistochemistry (IHC) kit (ab64259, Abcam). Tissue sections were air-dried for 2 h at room temperature, placed in a 37°C incubator overnight, deparaffinized in xylene, and rehydrated through alcohol gradients. Endogenous peroxidase was blocked with the Peroxidase Blocking Solution provided with the IHC kit. The sections were immersed in prewarmed 10 mM Na-citrate buffer (freshly prepared, pH 6.0) and kept in a boiling water bath for 20 min (antigen retrieval). After antigen retrieval, sections were rinsed in PBST (PBS with 0.01% Tween-20) followed by BSA blocking (5% wt/vol in PBST) for 1 h at room temperature to reduce nonspecific binding of the antibodies. The primary anti-Parp14 antibody in BSA (5% wt/vol in PBST) was added to tissue sections and incubated overnight at 4°C in a humidified chamber. Post-incubation with primary antibody, tissue sections were rinsed twice in PBST for 5 min each and incubated with biotinylated anti-mouse secondary antibody (provided with IHC kit) for 1 h at room temperature. Streptavidin-HRP conjugate was added and then stained using 3,3′-diaminobenzidine (DAB) as a chromogen (both solutions were provided with IHC kit). Harris hematoxylin was used as a nuclear counterstain. Sections without incubation of primary antibody served as negative controls. Mounting was performed using Histo-Clear, and sections were allowed to sit at room temperature for 12 h before imaging. Imaging was performed using Zeiss AxioImager M1 microscope with 5×, 20×, 40× oil or 63× oil objective lenses.

### Parp14 and F4/80 double immunofluorescence staining

Staining was performed as previously described (35). Briefly, Alexa Fluor 647 conjugated anti-F4/80 (MCA497A647, Bio-Rad, dilution 1:500) and anti-Parp14 (sc-377150, Santa Cruz Biotechnology, dilution 1:500) were mixed in 5% (wt/vol) BSA in PBST. Secondary antibody Alexa Fluor 488 goat anti-mouse IgG (H+L) (A11001, Invitrogen, dilution 1:1,000) was used to visualize the anti-Parp14 antibodies. Mounting was performed using Histo-Clear post-DAPI counterstaining, and sections were kept in the dark at 4°C before Zeiss AxioImager M1 imaging.

### Parp14 and wheat germ agglutinin double immunofluorescence staining

After antigen retrieval (as explained above), sections were permeabilized using 0.2% Triton X-100 in PBS for 10 min at room temperature. This was followed by BSA blocking (5% wt/vol in PBST) for 1 h at room temperature. Primary anti-Parp14 antibody in BSA (5% wt/vol in PBST) staining was performed as mentioned above. After overnight incubation, tissue sections were rinsed in PBST twice for 5 min each and incubated with secondary antibody Alexa Fluor 647 goat anti-mouse IgG (A21235, Invitrogen, dilution 1:1,500) for 1 h at room temperature. After secondary antibody incubation, tissue sections were rinsed in PBST thrice for 5 min each and incubated with Alexa Fluor 488-conjugated wheat germ agglutinin (W11261, Invitrogen, dilution 5 µg/mL) in PBS for 10 min at room temperature. Tissues were then washed twice in PBST for 5 min each, and mounting was performed using Mowiol (475905, EMD Millipore) with 2.5% wt/vol DABCO (1,4-diazabicyclo[2.2.2]octane, D27802, Sigma) after DAPI counterstaining. Sections were kept in the dark for 1 h at room temperature, then stored at 4°C before Zeiss AxioImager M1 imaging.

### QuPath-based quantitation of Parp14 immunohistochemical staining

The anti-Parp14-stained tissue sections of wt mice (distal small intestine, cecum, and proximal large intestine) were scanned using a Pannoramic 1000 Slide scanner (3DHistech, Budapest, Hungary) with a 40× objective and analyzed with a Pannoramic Viewer (3DHistech, software version 1.15.4). Scanned slides were converted to .mrxs file type, and a specific project in QuPath (qupath.github.io, software version 0.5.1 [36]) was created to quantify the DAB OD (optical density) of the entire cell, the cytosol, and the nucleus. Parameters for stain vectors of hematoxylin and DAB were set using automatic estimation in QuPath. For cellular detection, nucleus parameters used were background radius, 8 µm; minimum area, 10 µm^2^; and maximum area, 400 µm^2^. Cell expansion was set at 5 µm. From each tissue of each section, a minimum of 50 to a maximum of 200 horizontal villus crypts were selected for cellular detection and for quantifying DAB staining intensity from manually annotated regions of interest consisting mostly of epithelial cells. Detected annotations were exported, and statistical analyses were conducted using the one-way ANOVA with Tukey’s multiple-comparison test to compare the means.

### Single-cell RNA-Seq

The already published single-cell RNA-Seq data on epithelial cell-enriched cell suspensions of control (four mice) vs *S. Typhimurium*-infected (SL1344 strain, 2 days post-infection, two mice) C57BL/6J mice (37) were re-analyzed. We used the single-cell RNA-Seq data analysis and visualization interface at the Broad Institute Single Cell Portal (https://singlecell.broadinstitute.org/single_cell) in order to analyze *Parp1* and *Parp14* expression. In respect for the comparative data analysis with our bulk tissue RNA-Seq data, we first downloaded the differential single-cell RNA-Seq gene expression data (Table S9 of reference 37). Next, we filtered the single-cell RNA-Seq data to only include differentially expressed genes with a <0.05 statistical support value (FDR-corrected Mann-Whitney *P*-value). Subsequently, we executed two comparisons, that is, (i) genes upregulated by infection in wt mice (single-cell data) vs genes downregulated by infection in Parp14-deficient mice (bulk tissue data), and (ii) genes downregulated by infection in wt mice (single-cell data) vs genes upregulated by infection in Parp14-deficient mice (bulk tissue data).

### Isolation of RNA and quantitative PCR analysis

Total RNA was isolated from mouse tissues using TRIsure reagent (BIO-38033, Bioline GmbH, Germany), and genomic DNA was digested using RNase-free DNase (rDNase) from Machery-Nagel as per the manufacturer’s instructions. Briefly, colon tissue was homogenized using stainless steel balls (IKA 5 mm stainless steel balls, Fisher Scientific) and compact bead mill (TissueLyser LT, Qiagen). Colon tissue was placed in TRIsure reagent during homogenization. After this, chloroform was used for phase separation. RNA in the upper aqueous phase was precipitated using isopropyl alcohol. The pellet was washed with 70% ethanol and dissolved in nuclease-free water. Dissolved RNA was mixed with rDNase and rDNase reaction buffer as per manufacturer’s instructions. This mixture was incubated at 37°C for 10 min. Post-gDNA digestion, RNA was precipitated using 3 M sodium acetate, pH 5.2, and 96% ethanol. Pellet was washed with 70% ethanol and dissolved in RNase-free water. RNA purity and concentration were measured using DeNovix DS-11 spectrophotometer (Wilmington, DE, USA). The dissolved RNA (1 µg) was reverse-transcribed using SuperScript III reverse transcriptase (#1808044, Thermo Fisher Scientific) and Oligo(dT) 12–18 Primer (#18418012, Thermo Fisher Scientific). Separate real-time PCR was carried out in duplicates using TaqMan gene expression assays (Applied Biosystems) for *Parp14* (Assay ID: Mm00520984_m1), *Il1b* (Assay ID: Mm00434228_m1), *Il6* (Assay ID: Mm00446190_m1), *Ccl2* (Assay ID: Mm00441242_m1), *Ccl7* (Assay ID: Mm00443113_m1), *Tnfa* (Assay ID: Mm00443258_m1), *Il23a* (Assay ID: Mm00518984_m1), *Il17a* (Assay ID: Mm00439618_m1), *Cxcl1* (Assay ID: Mm04207460_m1), *Cxcl2* (Assay ID: Mm00436450_m1), *Cxcl10* (Assay ID: Mm00445235_m1), *Spink1* (Assay ID: Mm00436765_m1), *Sst* (Assay ID: Mm00436671_m1), *ApoA1* (Assay ID: Mm00437569_m1), *Ckb* (Assay ID: Mm00834780_g1) and the reference gene, glyceraldehyde-3-phosphate dehydrogenase, *Gapdh* (Assay ID: Mm99999915_g1) on the Rotor-Gene Q real-time PCR cycler (Qiagen). Thermal cycling conditions included an initial denaturation step at 95°C for 10 min followed by 40 cycles of 95°C for 15 s and 60°C for 1 min. Relative mRNA levels were determined using the 2^-ΔΔCT^ method with *Gapdh* as reference (38). If the standard deviation of duplicate Ct values from a sample was 0.5 or more, results of those samples were not used for statistical analysis. Statistical analysis of 2^-ΔΔCT^ values was done using the unpaired *t*-test (two-tailed).

### RNA-Seq runs and data analysis

Total RNA of the distal colon tissue samples was extracted as described above.

#### RNA quality

RNA integrity, including all the other RNA-Seq wet-lab techniques described below, was assessed by Novogene Co., Ltd. (Cambridge, UK) using the RNA Nano 6000 Assay Kit on the Bioanalyzer 2100 system (Agilent Technologies, CA, USA).

#### Library preparation for transcriptome sequencing

Briefly, mRNA was purified from total RNA using poly-T oligo-attached magnetic beads. Fragmentation was carried out using divalent cations under elevated temperature in First Strand Synthesis Reaction Buffer (5×). First-strand cDNA was synthesized using random hexamer primers and M-MuLV Reverse Transcriptase (RNase H-). Second-strand cDNA synthesis was subsequently performed using DNA Polymerase I and RNase H. Remaining overhangs were converted into blunt ends via exonuclease/polymerase activities. After adenylation of 3′ ends of DNA fragments, adaptors with a hairpin loop structure were ligated to prepare for hybridization. To select cDNA fragments preferentially 370–420 bp in length, the library fragments were purified using the AMPure XP system (Beckman Coulter, Beverly, USA). Then PCR was performed with Phusion High-Fidelity DNA polymerase, Universal PCR primers, and Index (X) Primer. Finally, PCR products were purified (AMPure XP system), and library quality was assessed on the Agilent Bioanalyzer 2100 system.

#### Clustering and sequencing

The clustering of the index-coded samples was performed on a cBot Cluster Generation System using TruSeq PE Cluster Kit v.3-cBot-HS (Illumina) according to the manufacturer’s instructions. After cluster generation, the library preparations were sequenced on an Illumina NovaSeq platform, and 150 bp paired-end reads were generated. The raw RNA-Seq data have been deposited in the NCBI Gene Expression Omnibus database (https://www.ncbi.nlm.nih.gov) under accession number GSE284287.

#### Data analysis - Quality control

Raw data (raw reads) of FASTQ format were first processed through Novogene’s in-house Perl scripts. In this step, clean data (clean reads) were obtained by removing reads containing adapters, reads containing poly-N, and low-quality reads from raw data. At the same time, Q20, Q30, and GC content of the clean data were calculated.

#### Data analysis - reads mapping to the reference genome

Reference genome and gene model annotation files were downloaded directly from the genome website. The index of the reference genome was built using Hisat2 v.2.0.5, and paired-end clean reads were aligned to the reference genome using Hisat2 v.2.0.5.

#### Data analysis - quantification of transcript level

FeatureCounts v.1.5.0-p3 was used to count the read numbers mapped to each gene. Subsequently, fragments per kilobase of transcript sequence per millions base pairs sequenced (FPKM) for each gene were calculated based on the length of the gene and the number of reads mapped to that gene.

#### Data analysis - differential expression analysis

Differential expression analysis was performed using the DESeq2 R package (v.1.20.0). The resulting *P*-values were adjusted using the Benjamini and Hochberg approach for controlling the false discovery rate. Genes were assigned as differentially expressed either with low (*P*-value <0.05) or high statistical stringency (adjusted *P*-value, *P*_adj_ < 0.05).

#### Data analysis - GO and KEGG analyses

To analyze cellular and physiological associations of the uniquely expressed genes (FPKM > 1) and the differentially expressed genes (DEGs), we performed Gene Ontology (GO) enrichment at the GO consortium website (https://geneontology.org) (39–41). In parallel, we performed the Kyoto Encyclopedia of Genes and Genomes (KEGG) pathway analysis (42–44) using SRplot (45) (http://www.bioinformatics.com.cn/srplot).

## RESULTS

### Parp14 is expressed by epithelial cells in the mouse gastrointestinal tract

**Fig 1:**
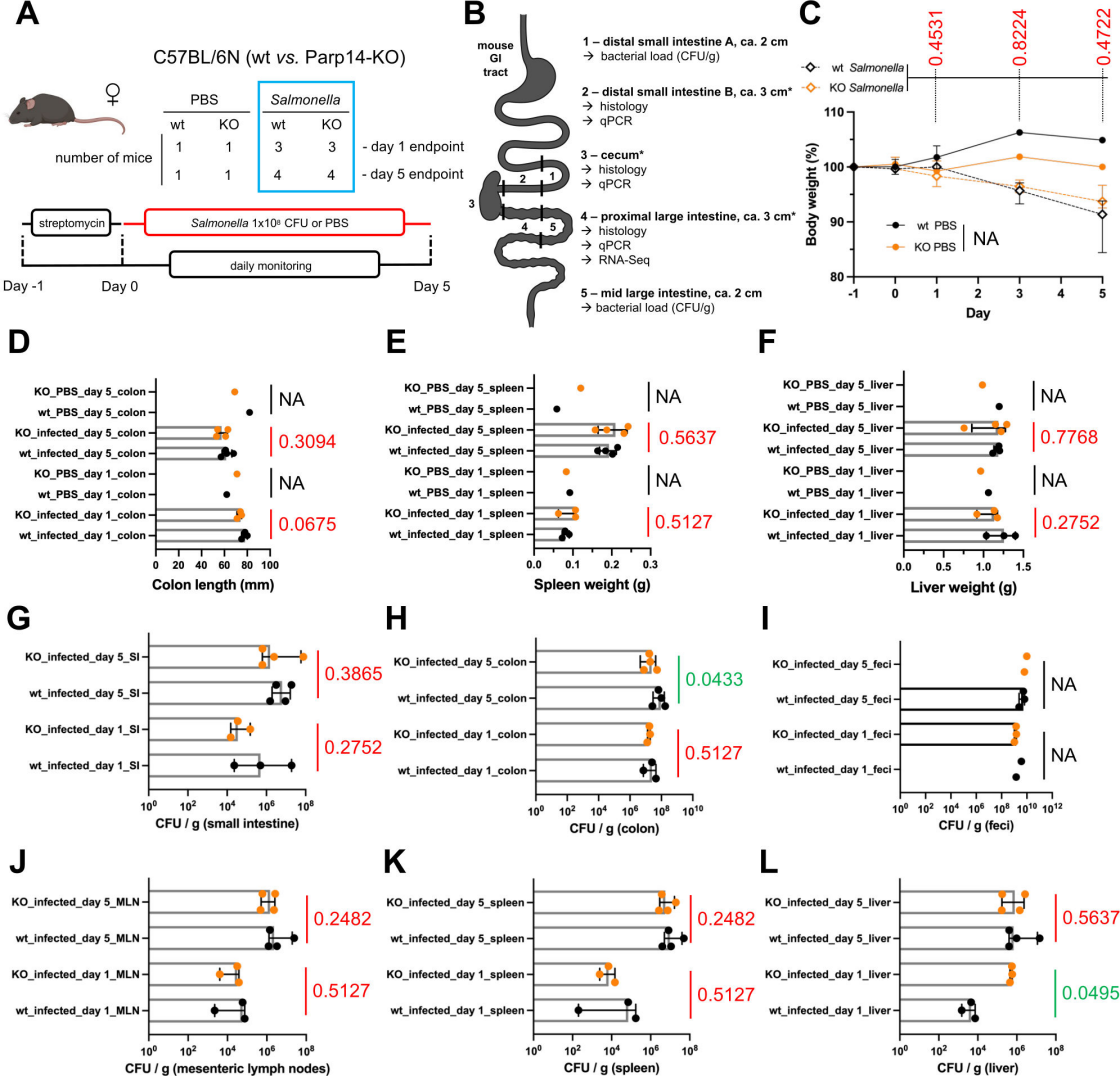
Minor macroscopic effects of Parp14 deficiency on the severity of salmonellosis. (**A, B**) Schematic representation of the single-animal experiment executed in this study. The blue box refers to mice, which were subjected to statistical comparisons throughout the study. The tissues marked with an asterisk were longitudinally cut into two pieces, one for histology and one for qPCR/RNA-Seq. Images were partially created with BioRender.com. (**C**) Weight change of the mice during the course of the experiment relative to day −1 (medians with interquartile range). No statistically significant differences between the infected wt and Parp14-deficient mice were detected. Statistical significance values are shown in the figure. Weights of the PBS mice were not statistically compared (NA, not applicable; fewer than three animals to compare, see Fig. 1A). (**D**) Colon lengths at day 1 and day 5. (**E**) Spleen weights at day 1 and day 5. (**F**) Liver weights at day 1 and day 5. (**G–L**) Determination of viable bacteria in different tissues at day 1 and day 5. Bars in sub-panels D–L represent medians with interquartile range. All individual data points are shown. Statistical significance values for the differences between the infected wt and Parp14-deficient mice are shown in each D–L sub-panel. Fecal pellets were not obtained from all mice. Parameters of the PBS mice were not statistically compared (NA, not applicable; fewer than three animals to compare, see Fig. 1A).

**Fig 2:**
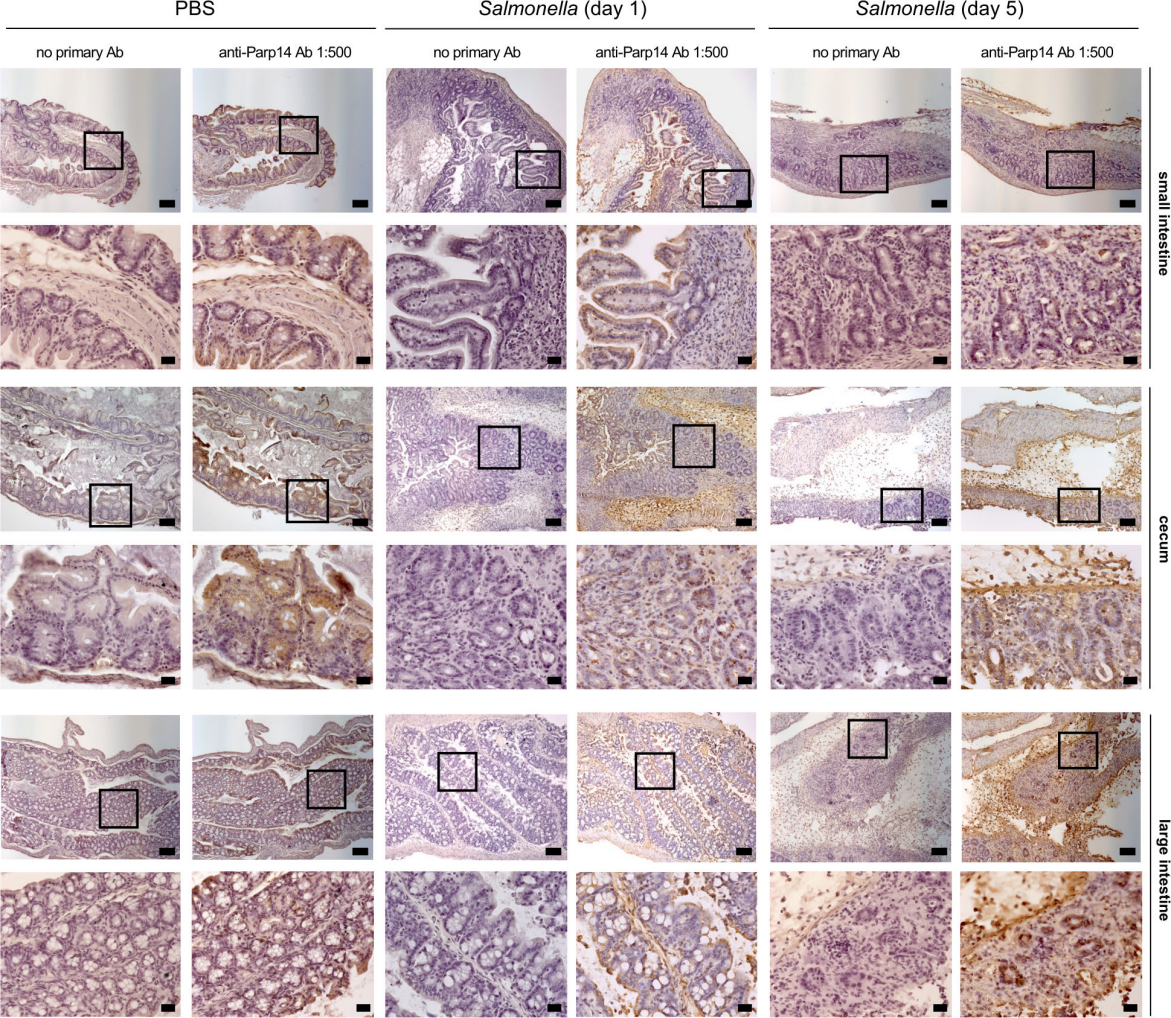
Immunohistochemical staining of Parp14 in the mouse gastrointestinal tract. Parp14 was detected in FFPE tissue sections using a commercial anti-Parp14 antibody. Selected representative 10× air (upper image, scale bar: 100 µm) and 40× oil (lower image, scale bar: 20 µm) images are shown for each location of the gastrointestinal tract (1:500 dilution of the anti-Parp14 antibody). Quantitation of Parp14 staining is displayed in Fig. 3 and Fig. S3.

**Fig 3:**
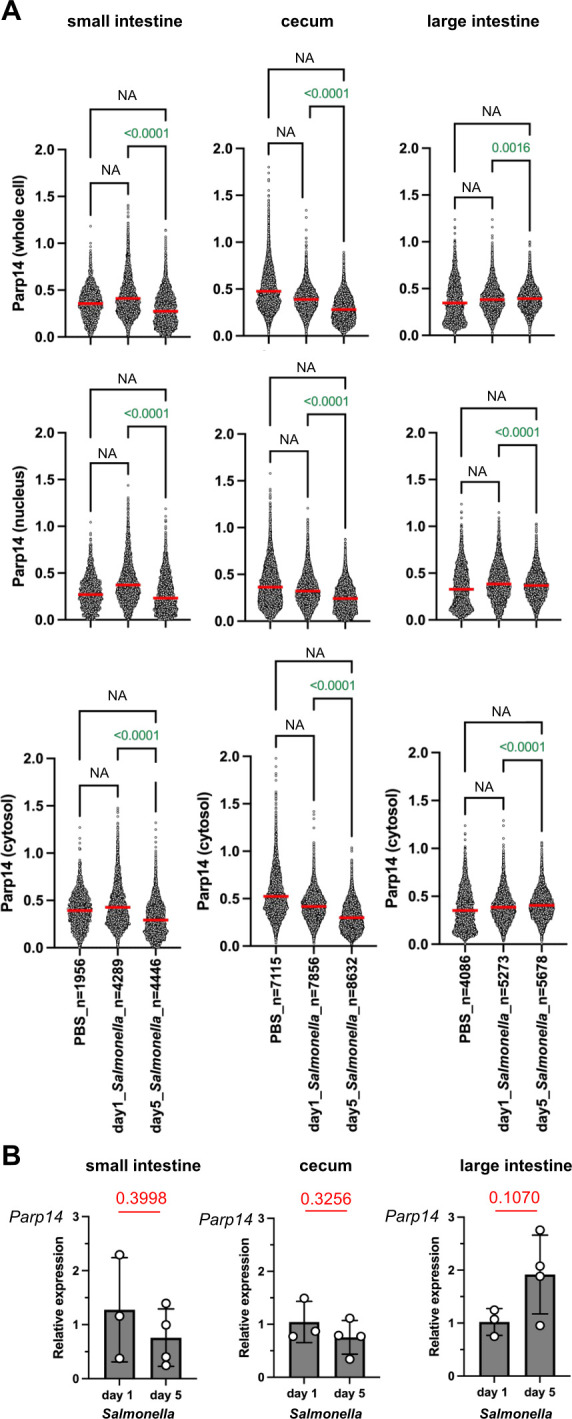
Quantitation of Parp14 expression in the mouse gastrointestinal tract. (**A**) The QuPath-based quantitation of Parp14 expression. Representative examples of the Parp14 stainings are shown in Fig. 2. The values on the *y*-axis refer to the means of DAB staining intensity, that is, the mean OD in the QuPath data output. Each dot refers to a single cell. The numbers of analyzed cells (mostly epithelial cells) are indicated on the *x*-axis (see Fig. S3). The red lines above the data points refer to the mean values. Statistical analyses were conducted using the two-tailed unpaired *t*-test (NA, not applicable; fewer than three animals to compare, see Fig. 1A). One *Salmonella*-infected day 5 mouse was left out from the quantitation due to poor quality of the FFPE tissue block. (**B**) The qPCR data on relative *Parp14* expression (means with standard deviation, statistics performed using two-tailed unpaired *t*-test). Samples were included in the data analysis if they passed the 0.5 standard deviation Ct filter for replicate runs. No statistical analyses were executed against the PBS groups because there were less than three data points/animal to compare (see Fig. 1A). The calibrators in each sub-panel are the mean dCq values of the day 1 *Salmonella*-infected mice.

Littermates of female wt and Parp14-deficient mice (34, 35) were either orally gavaged with *S*. Typhimurium strain SL1344 or PBS, followed by sampling as described in Fig. 1A and B. First, we analyzed the expression of Parp14 at the protein level in wt mice using a commercial anti-Parp14 monoclonal antibody. This antibody was validated for epitope specificity in our previous study (35). We detected Parp14-positive epithelial cells across the mucosal tissues in small intestine, cecum, and large intestine of *S*. Typhimurium- as well as PBS-gavaged mice (Fig. 2; Fig. S1). The mucosal tissue also contained some Parp14-positive macrophages (F4/80-positive cells), as exemplified with the large intestine analysis (Fig. S2). A QuPath-based quantitation of the Parp14 staining intensity was executed by selecting 50–200 horizontal villus cross-sections and thereby thousands of individual epithelial cells per animal (Fig. S3). This analysis revealed a temporal pattern of Parp14 staining (Fig. 3A). The Parp14 staining in the small intestine appeared to increase with infection at day 1 but decreased from day 1 to day 5 (*P*<0.0001). The Parp14 staining in the cecum appeared to decrease with infection at day 1 and decreased from that level further down at day 5 (*P*<0.0001). The large intestine showed more complex staining patterns. The Parp14 staining appeared to increase with infection at day 1 and increased from that level further up at day 5 when the whole-cell or cytosolic QuPath readouts were compared (*P*<0.0001). However, less Parp14 staining was detected in the nucleus at day 5 as compared to day 1 (*P*<0.0001). Next, we conducted a qPCR-based quantitation of *Parp14* expression. We did not detect statistically significant differences between the two time points (Fig. 3B). We also analyzed the published small intestine single-cell RNA-Seq data on epithelial cell-enriched cell suspensions (Epcam+/CD45−/TER−119−/CD31−) of PBS control and *S*. Typhimurium (SL1344 strain, 2 days post-infection)-infected C57BL/6J mice (37). Out of the eight detected small intestine epithelial cell sub-types (endocrine cells, enterocytes, enterocyte progenitors, goblet cells, stem cells, transit-amplifying cells, early transit-amplifying cells, Tuft cells), the expression of *Parp14* was pronounced in the enterocytes and Tuft cells of the infected mice (Fig. S4). Such a pattern of expression was not detected in the PBS gavaged mice. Of note, the expression pattern of the founding member of the Parp family, *Parp1* (16), was similar in the eight epithelial cell types of *S*. Typhimurium and PBS gavaged mice, being most pronounced in the endocrine cells. Taken together, Parp14 is expressed by epithelial cells across the mucosal tissue in the mouse gastrointestinal tract.

### Minor macroscopic effects of Parp14 deficiency on the severity of salmonellosis

We monitored the *S*. Typhimurium- and PBS-gavaged mice daily by quantifying their body weight up to the day 5 termination point. Infection caused weight loss in wt and Parp14-deficient mice in a statistically similar manner (Fig. 1C). No differences between the infected wt and Parp14-deficient mice were detected in the colon length, spleen weight, or liver weight at day 1 or day 5 (Fig. 1D through F). However, when we quantified the numbers of viable bacteria in different tissues, some statistically significant differences were detected (Fig. 1G through L). The bacterial load (CFU/g) was higher in the liver of Parp14-deficient mice at day 1. In contrast, the bacterial load was lower in the colon of Parp14-deficient mice at day 5. Otherwise, no differences in the bacterial loads were detected. It appears, based on the measured macroscopic variables, that the effect of Parp14 deficiency on the severity of salmonellosis is minor.

### Exacerbated gastrointestinal histopathology in *S*. Typhimurium-infected Parp14-deficient mice

**Fig 4:**
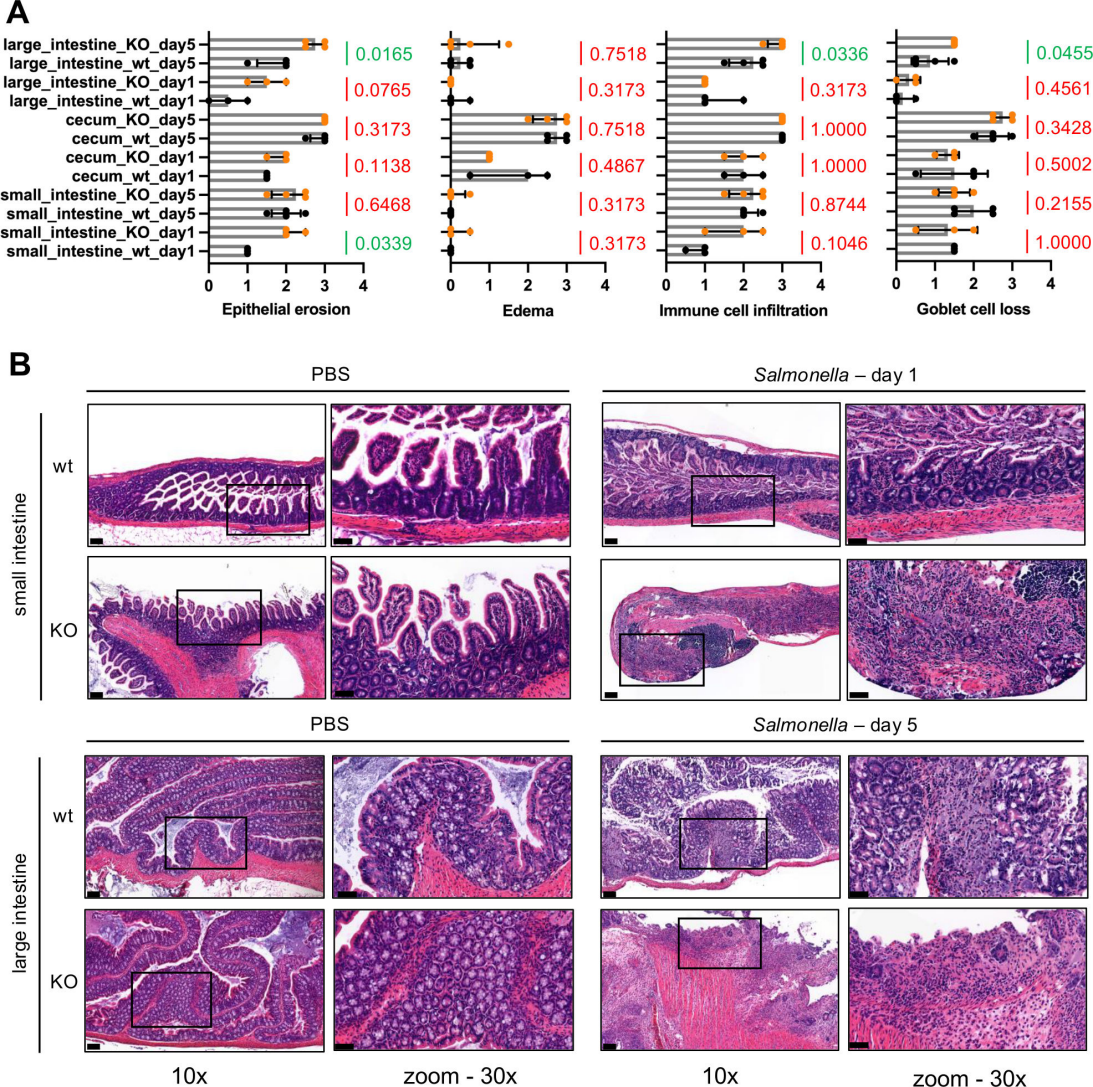
Exacerbated gastrointestinal histopathology in *S*. Typhimurium-infected Parp14-deficient mice. (**A**) Quantitation of histopathological variables, that is, epithelial erosion, tissue edema, immune cell infiltration, and goblet cell loss in distal small intestine, cecum, and large intestine (medians with interquartile range). Statistical significance values for the differences between infected wt and Parp14-deficient mice are shown in each sub-panel. (**B**) Representative HE-stained tissue sections. The 10× and 30× air objective images are shown. The size of the scale bar in sub-panels refers to 100 µm (10×) and 50 µm (30×). The sections represent the tissues where statistically significant differences were detected between wt and Parp14-deficient mice. Representative HE-stained tissue sections of all the tissue sections at all time points are shown in Fig. S5 as 10× air objective images.

We quantified epithelial erosion, edema, immune cell infiltration, and goblet cell loss in small intestine, cecum, and large intestine from the HE-stained FFPE sections (Fig. 4A and B; Fig. S5). Of note, the absence of Parp14 did not cause apparent small intestine, cecum, or large intestine deformations in the resting state, as evidenced with the PBS-gavaged mice (Fig. 4B). This is in line with our previous comparative results with water-gavaged wt and Parp14-deficient male mice (35). Several statistically significant histological differences between the infected wt and Parp14-deficient were detected (Fig. 4A). At day 1, epithelial erosion was stronger in the small intestine of Parp14-deficient mice. At day 5, epithelial erosion was also stronger in the large intestine of Parp14-deficient mice. Such a trend in the large intestine was evident already at day 1 (*P* = 0.0765). Moreover, at day 5, immune cell infiltration and goblet cell loss were stronger in the large intestine of Parp14-deficient mice. It appears that Parp14 deficiency caused a more severe salmonellosis in mice, as evidenced by the exacerbated histopathology, in particular in the large intestine.

### Transcriptomic signatures uniquely detected in large intestines of *S*. Typhimurium-infected wt and Parp14-deficient mice

**Fig 5:**
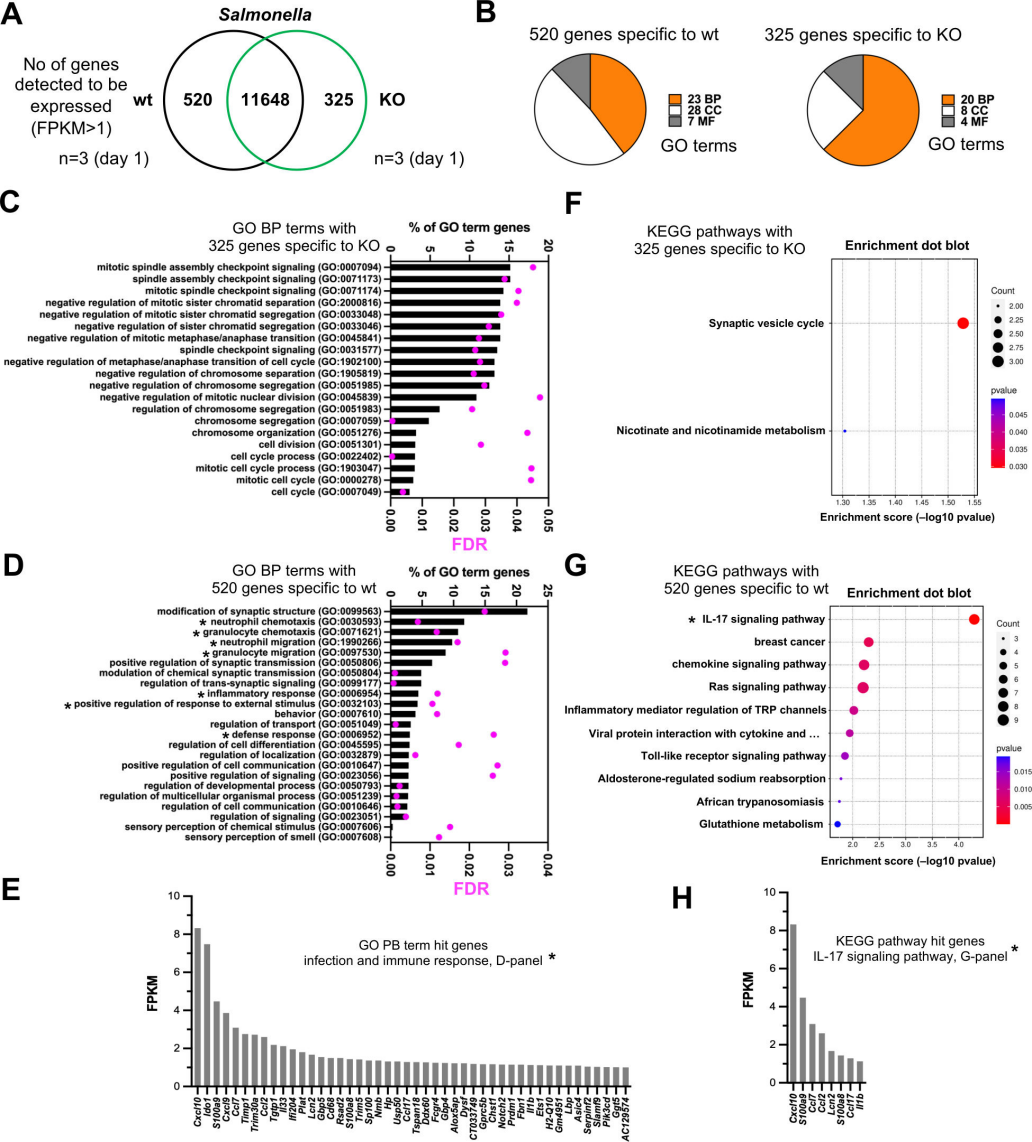
Transcriptional signatures uniquely detected in *S*. Typhimurium-infected wt and Parp14-deficient mice. Data from a triplicate RNA-Seq analysis of mouse large intestine sections 1 day post-infection are shown. (**A**) The Venn diagrams of shared and unique genes that were detected to be expressed in the infected wt and Parp14-deficient mice (FPKM value >1). The integer is the number of genes detected to be expressed in both of the genotypes. (**B**) The pie charts of the numbers of identified GO terms based on the genotype-specific lists of expressed genes (BP, biological process; CC, cellular component; MF, molecular function; Data Set S1). (**C–E**) Bar graph representation of all the identified GO BP terms with the genotype-specific lists of expressed genes. The BP terms are sorted based on the percentage of GO term gene values (number of detected genes in a particular BP term / number of all genes in particular BP term × 100). FDR refers to the false discovery rate value. An FDR value cut-off of <0.05 was used in the searches. The asterisks in the wt sub-panel (**D**) refer to the seven infection- and inflammation response-related BP terms. The sub-panel E displays the genes of these seven infection- and inflammation response-related BP terms. (**F–H**) Pathway-enrichment dot plot representations of all (KO sub-panel) and the top 10 (wt sub-panel) KEGG pathways identified with the genotype-specific lists of expressed genes. All the identified KEGG pathways with the corresponding gene lists are described in Data Set S1_11 and 12. The KEGG pathways are sorted based on the *P*-value. The count values refer to the number of genes that were detected in a particular KEGG pathway. The asterisk in the wt sub-panel (**G**) refers to the only KEGG pathway with a <0.05 *P*_adj_-value. The sub-panel H displays the genes of this IL-17 signaling pathway.

**Fig 6:**
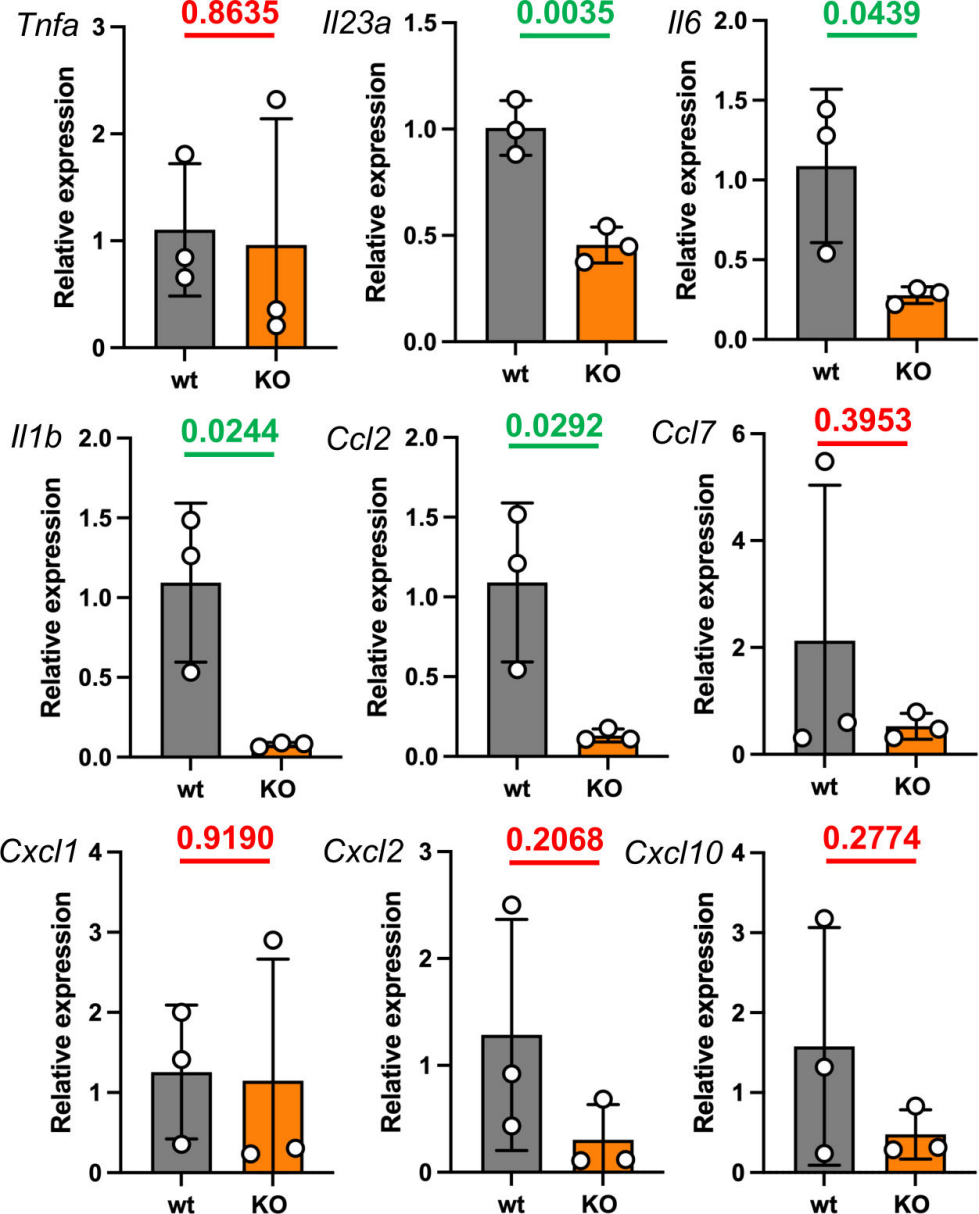
Hampered expression of four cytokines in the large intestine of *S*. Typhimurium-infected Parp14-deficient mice. Four hit genes of the large intestine bulk tissue RNA-Seq analysis (*Ccl2*, *Ccl7*, *Cxcl10*, *Il1b*) were analyzed. Five other TaqMan qPCR assays on inflammation-associated genes were run in parallel. The figure illustrates the TaqMan qPCR data on relative gene expression with means and standard deviations. The calibrators in all sub-panels are the mean dCq values of the day 1 infected wt mice. Statistical analyses were done with a two-tailed unpaired *t*-test. All the statistical significance values of the comparisons between the wt and Parp14-deficient mice are indicated.

We performed a bulk tissue large intestine RNA-Seq analysis using triplicate samples of infected wt and Parp14-deficient mice at day 1. First, we looked at the identities of genes detected to be expressed using the canonical transcript detection cut-off FPKM value of >1. As shown in Fig. 5A and Data Set S1_1–3, 11,648 genes were detected in both genotypes, as well as 520 and 325 genes specifically in the wt and Parp14-deficient mice, respectively. Next, we ran GO term searches with the wt and Parp14-deficient mouse-specific gene detections using the Fisher’s Exact test and the False Discovery Rate (FDR) <0.05 filter (Fig. 5B; Data Set S1_7 and 8). When we looked at the GO Biological Process (BP) terms, identified based on the 325 genes specifically detected in the Parp14-deficient mice, all the 20 BP terms were related to cell division (Fig. 5C). Altogether, 201 genes out of the 325 analyzed genes were mapped to these BPs (Data Set S1_9). When we ran the GO term analysis with the 520 genes specifically detected in the wt mice, 23 BP terms were identified (Fig. 5D). Seven of these BP terms had relevance to infection and inflammation responses, for example, neutrophil chemotaxis and leukocyte migration. Altogether, 46 genes were behind these seven BP term identifications, for example, cytokines *Ccl17*, *Ccl7*, *Ccl2*, *Cxcl9,* and *Cxcl10* (Fig. 5E; Data Set S1_10). The *Ccl2, Ccl7,* and *Cxcl10* genes were also analyzed in a TaqMan qPCR assay. We found out that the relative mRNA level of *Ccl2*, but not *Ccl7* or *Cxcl10*, was significantly higher in the large intestine of wt mice at day 1 (Fig. 6). Such a *Ccl2* mRNA-level difference was not detected with day 5 samples of the large intestine or with any of the small intestine or cecum samples (Fig. S6). When the gene lists of the seven infection- and inflammation response-related GO BP terms were compared with the PBS-gavaged wt mice gene lists (Data Set S1_4–6, 13, and 14), we found that four genes were shared (*Hp*, *Gprc5b*, *Trim30a,* and *H2-Q10*). Although we only had two PBS-gavaged mice per genotype, the data indicate that the unique detection of most of the 46 genes in the *S*. Typhimurium-gavaged wt mice was indeed associated with the infection, not merely with the mouse genotype difference. Next, we ran KEGG pathway analyses using the wt and Parp14-deficient mouse-specific gene detections in *S*. Typhimurium infection. Based on the canonical <0.05 *P*-value filtering, only two KEGG pathways were detected with the Parp14-deficient mouse-specific gene list, and none were significant based on the <0.05 *P*_adj_-based filtering (Fig. 5F, Data Set S1_11). In contrast, based on the canonical <0.05 *P*-value filtering, we identified 20 KEGG pathways with the wt-specific genes (Fig. 5G; Data Set S1_12), and one of these was significant based on the <0.05 *P*_adj_ based filtering. This *P*_adj_ significant KEGG pathway was the IL-17 pathway containing altogether eight scored genes (*Lcn2*, *Il1b*, *S100a8*, *S100a9*, *Cxcl10*, *Ccl17*, *Ccl7*, *Ccl2*) (Fig. 5H). The *Il1b* gene was also analyzed in a TaqMan qPCR assay. We found out that the relative mRNA level of *Il1b* gene was significantly higher in the wt mice (Fig. 6). Such an *Il1b* mRNA-level difference was not detected with day 5 samples of the large intestine or with any of the small intestine or cecum samples (Fig. S6). Taken together, based on the FPKM transcript-level analysis, we detected a cell division-related transcriptomic signature in Parp14-deficient mice that was missing from the wt mice in *S*. Typhimurium infection. Also, we detected and validated an infection- and inflammation response-related transcriptomic signature in the wt mice that was missing from the Parp14-deficient mice in *S*. Typhimurium infection.

### Transcriptomic signatures downregulated in the large intestine of *S*. Typhimurium-infected Parp14-deficient mice

**Fig 7:**
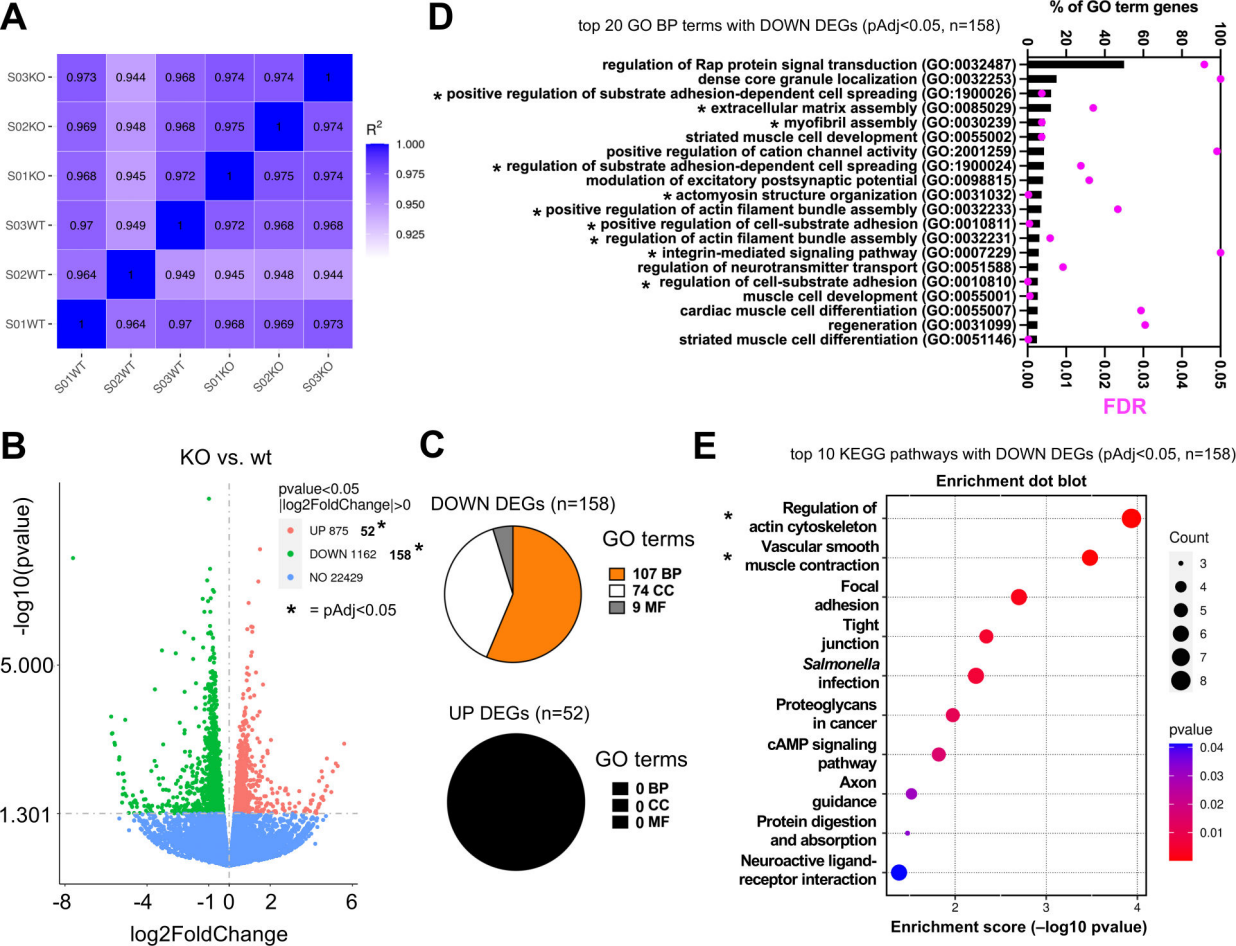
Transcriptional signature downregulated in *S*. Typhimurium-infected Parp14-deficient mice. Data from triplicate bulk tissue RNA-Seq analysis of mouse large intestine sections 1 day post-infection are shown. (**A**) Inter-sample correlation heatmap based on the FPKM values of the DEGs in Parp14-deficient vs wt mice comparison. *R*^2^ is the square of Pearson correlation coefficient (*R*). (**B**) Volcano plots of the DEGs. Specific information on the DEGs is given in Data Set S1_13 and 14. The *x*-axis shows the fold difference in gene expression between different samples, and the *y*-axis shows the statistical significance of the differences. Red dots represent upregulation genes, and green dots represent downregulation genes. The dashed line indicates the threshold line for statistically significant differential gene expression. The values marked with asterisks refer to the number of DEGs that were used for a stringent downstream data analysis, that is, UP genes, log2(FoldChange) > 0.5 and *P*_adj_ < 0.05; DOWN genes, log2(FoldChange) < −0.5 and *P*_adj_ < 0.05 (Data Set S1_14). (**C**) GO term analysis with DEGs in Parp14-deficient vs wt mice comparison (BP, biological process; CC, cellular component; MF, molecular function; Data Set S1_15). The GO terms were searched using the canonical Fisher’s test and an FDR value <0.05 filter. (**D**) Bar graph representations of the top 20 identified GO BP terms (all the 107 identified GO BP terms in Data Set S1_15) sorted based on the percentage of GO term gene values (number of detected genes in a particular GO term / number of all genes in a particular GO term × 100). The black asterisks in the sub-panel refer to the PB terms with functional relevance to cell adhesion and cytoskeleton remodeling. (**E**) Pathway-enrichment dot plot representations of the top 10 identified KEGG pathways sorted based on the *P*-value. All the identified KEGG pathways with the corresponding gene lists are described in Data Set S1_16. The count values refer to the number of genes that were detected in a particular KEGG pathway. The black asterisk in the wt sub-panel refers to the KEGG pathways with a <0.05 *P*_adj_-value.

**Fig 8:**
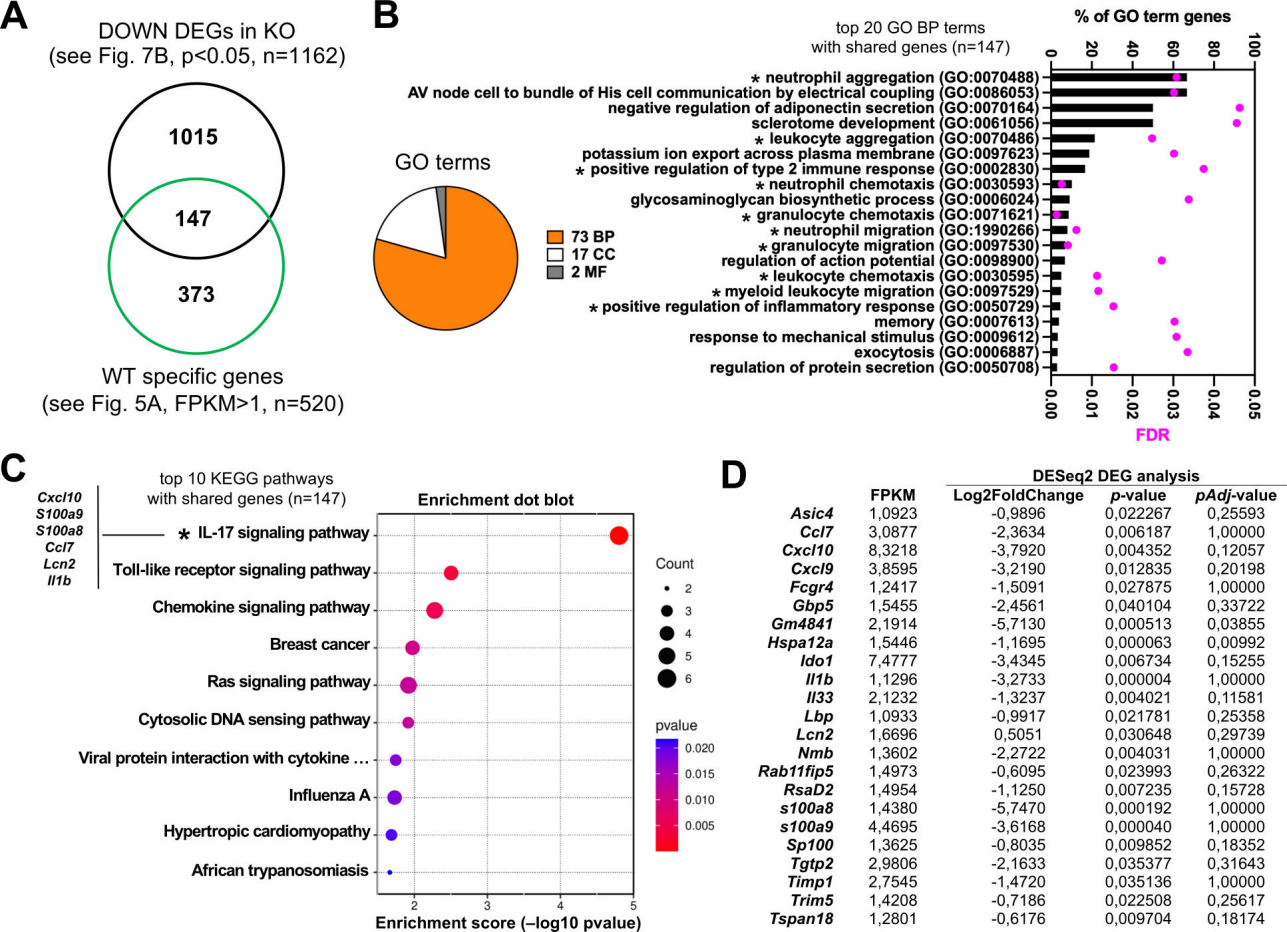
Comparative analysis of unique and differentially expressed transcriptional signatures. (**A**) The Venn diagrams of the shared and unique genes of the FPKM analysis vs the DESeq2 DEG analysis. The integer is the number of genes that were uniquely detected in the *S*. Typhimurium-infected wt mice based on the FPKM analysis and the genes that were downregulated in the *S*. Typhimurium-infected Parp14-deficient mice based on the DESeq2 analysis. (**B**) The pie chart- and bar graph-based representation of the identified GO terms (BP, biological process; CC, cellular component; MF, molecular function) with the 147 shared genes (see also Data Set S1_19). The top 10 BP terms are shown, and they are sorted based on the percentage of GO term gene values (number of detected genes in a particular BP term / number of all genes in particular BP term × 100). FDR refers to the false discovery rate value. An FDR cut-off value of <0.05 was used in the searches. The asterisks refer to the infection- and inflammation response-related BP terms. (**C**) Pathway-enrichment dot plot representations of the top 10 KEGG pathways identified with the shared genes (see also Data Set S1_20). The KEGG pathways are sorted based on the *P*-value. The count values refer to the number of genes that were detected in a particular KEGG pathway. The asterisk refers to the only KEGG pathway with a <0.05 *P*_adj_-value. (**D**) The key metrics of all the genes behind the 15 infection- and inflammation response-related GO BP terms and the KEGG IL-17 signaling pathway identifications.

Next, we executed a DESeq2-based DEG analysis with triplicate groups of infected Parp14-deficient vs wt mice at day 1 (Data Set S1_15). The DESeq2-based DEG approach uses a different bioinformatic approach on the same raw RNA-Seq data output as in the preceding chapter’s FPKM transcript-level analysis. The pairwise *R*^2^ Pearson correlation values of the FPKM values of the DEGs were 0.974 or higher with the Parp14-deficient group, and 0.964 or higher with the wt group (Fig. 7A). This indicates that the triplicate samples could be reliably pooled for further analysis. The volcano plot of all the DEGs (*P* < 0.05) is shown in Fig. 7B. To execute downstream analysis with these DEGs, we ran the DEGs through a *P*_adj_ < 0.05 filter, which resulted in a shorter list of DEGs, that is, 52 upregulated DEGs and 158 downregulated DEGs (Data Set S1_16). When we performed a GO analysis with the upregulated DEGs, that is, the genes that had more transcripts in the knockout as compared with the wt mice, using the canonical Fisher’s test and FDR < 0.05 filter, we did not detect a single BP, CC, or MF GO terms (Fig. 7C). In contrast, with downregulated DEGs, that is, the genes that had fewer transcripts in the knockout as compared with the wt mice, numerous BP, CC, and MF GO terms were detected (Data Set S1_17; Fig. 7C and D). Many of the top downregulated DEG BP GO terms had functional relevance with cell adhesion and cytoskeleton remodeling (Fig. 7D). We also performed a KEGG pathway analysis with the downregulated DEGs. Based on the canonical <0.05 *P*-value filtering, we identified 12 KEGG pathways, and 2 of these were significant based on the <0.05 *P*_adj_ filtering (Fig. 7E; Data Set S1_18). The top *P*_adj_ significant KEGG hit was the regulation of actin cytoskeleton pathway containing altogether eight scored DEGs (*Ppp1r12b*, *Pfn2*, *Enah*, *Myh11*, *Fn1*, *Rdx*, *Myl9*, *Myh10*). Many of these DEGs were shared with the lower ranking >0.05 *P*_adj_ KEGG hits, including the focal adhesion and tight junction pathways (Data Set S1_18). It is noteworthy that infection- and inflammation response-related GO terms or KEGG pathways, similar to the FPKM transcript-level analysis (Fig. 5D and E and G and H), were not detected with the *P*_adj_ < 0.05 downregulated DEGs. We therefore dropped the statistical stringency of our DESeq2 analysis by switching the *P*_adj_ < 0.05 filter to the *P* < 0.05 filter. Next, we compared the overlap between the downregulated DEGs (*n* = 1,162, see Fig. 7B) and the genes (*n* = 520) detected to be expressed only in the wt (see Fig. 5A). It turned out that 147 genes were shared with these two bioinformatic outputs (Fig. 8A). We identified 73 BP GO terms with these 147 genes, and 15 of them had relevance to infection and inflammation responses (Fig. 8B; Data Set S1_19), for example, neutrophil chemotaxis and leukocyte migration. We also identified 17 KEGG pathways (Fig. 8C; Data Set S1_20), and 1, the KEGG IL-17 signaling pathway, was significant based on the <0.05 *P*_adj_ filtering. The key metrics of the genes behind the 15 infection- and inflammation response-related GO BP terms and the KEGG IL-17 signaling pathway identifications are shown in Fig. 8D. Taken together, based on the DESeq2 DEG analysis, we detected a downregulated cell adhesion and cytoskeleton remodeling-related transcriptomic signature in the *S*. Typhimurium-infected Parp14-deficient mice. Also, by lowering the statistical stringency of the DESeq2 DEG analysis, we detected, in analogy to the FPKM transcript-level analysis (see Fig. 5 and 6), downregulated infection and inflammation response transcriptomic signatures in *S*. Typhimurium-infected Parp14-deficient mice.

### Epithelial cell-specific transcriptomic signature downregulated in large intestine of *S*. Typhimurium-infected Parp14-deficient mice

**Fig 9:**
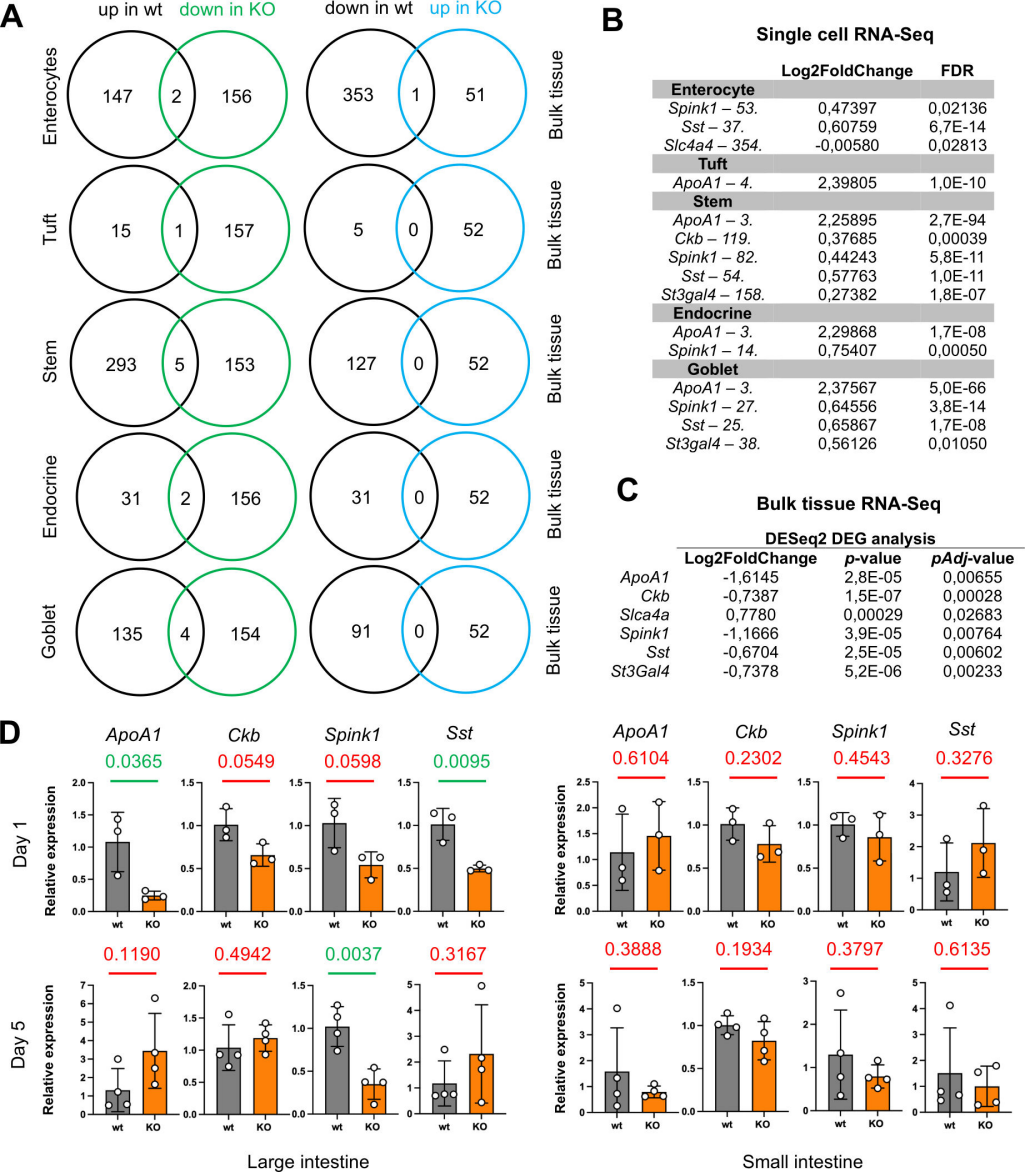
Epithelial cell-specific transcriptomic signature downregulated in the large intestine of *S*. Typhimurium-infected Parp14-deficient mice. (**A**) The Venn diagrams of the shared and unique genes in two comparisons, that is (i) genes upregulated by infection in wt mice (single-cell data [37]) vs genes downregulated by infection in Parp14-deficient mice (bulk tissue data), and (ii) genes downregulated by infection in wt mice (single-cell data [37]) vs genes upregulated by infection in Parp14-deficient mice (bulk tissue data). (**B**) The key single-cell RNA-Seq differential expression metrics of the shared genes. The numbers behind the gene names indicate the rank numbers, for example, *ApoA1* was the third highest upregulated gene in goblet cells. (**C**) The key bulk tissue differential expression metrics of the shared genes. (**D**) TaqMan qPCR validation of the four shared genes with small and large intestine samples at day 1 and day 5. The figure illustrates the TaqMan qPCR data on relative gene expression with means and standard deviations. The calibrators in all sub-panels are the mean dCq values of the infected wt mice. Statistical analyses were done with a two-tailed unpaired *t*-test. All the statistical significance values of the comparisons between the wt and Parp14-deficient mice are indicated.

Parp14 was expressed by epithelial cells across the mucosal tissue in the mouse gastrointestinal tract, that is, in the small intestine, cecum, and large intestine (see Fig. 2; Fig. S1). To experiment on the plausible epithelial cell functions of Parp14 in *S*. Typhimurium infection, we performed a comparative analysis of the published single-cell RNA-Seq data of small intestine epithelial cell types (37), which have counterparts in the large intestine, with our bulk large intestine RNA-Seq data. We executed two comparisons, that is, (i) genes upregulated by infection in wt mice (single-cell data) vs genes downregulated by infection in Parp14-deficient mice (bulk tissue data), and (ii) genes downregulated by infection in wt mice (single-cell data) vs genes upregulated by infection in Parp14-deficient mice (bulk tissue data). As shown in Fig. 9A through C, the latter comparison yielded one shared gene with the enterocyte input, that is, *Slc4a4*. However, several genes with multiple epithelial cell subtype inputs were identified in the first comparison (Fig. 9A through C). Four of these genes were analyzed in TaqMan qPCR assays. We found that none of these genes were differentially expressed in the small intestine of infected Parp14-deficient mice. However, when we analyzed samples of the large intestine, which, based on the histopathology analysis (see Fig. 4), suffered the most from Parp14 deficiency, differential gene expression was detected. The relative mRNA levels of *ApoA1* (day 1), *Spink1* (day 5), and *Sst* (day 1) were significantly lower in the infected Parp14-deficient mice (Fig. 9D). Taken together, a previously identified infection-induced epithelial cell-specific transcriptomic signature (37) was detected to be downregulated in the large intestine of *S*. Typhimurium-infected Parp14-deficient mice.

## DISCUSSION

The microbiota-mediated colonization resistance of incoming pathogens in the gastrointestinal tract is supported by thick intestinal mucous layer secreted by the epithelial cells (9, 10). If these barriers are breached, an innate inflammatory response is initiated, followed by the adaptive immune system activation, all designed to eradicate the invading pathogen. In some cases, such as in *S*. Typhimurium infection, the bacterium is thought to benefit from the colon inflammation to outcompete the colon lumen microbiota (13, 14), that is, via creation of gut dysbiosis, and to obtain nutrients boosting the colon luminal bacterial replication (15). It remains unknown at the molecular level how, and at which point, the mucosal inflammation turns anti-bacterial and how the gastrointestinal tissue homeostasis is restored.

We previously reported that the lack of Parp14 sensitized mice to dextran sulfate sodium (DSS)-induced colitis, manifested, for example, as stronger epithelial erosion compared to the wt mice (35). Oral administration of the non-physiological DSS chemical to mice via drinking water, used frequently to model human ulcerative colitis (46, 47), induces severe colitis characterized by weight loss, bloody diarrhea, loss of epithelial cells, and infiltration of neutrophils. DSS is believed to cause direct damage to epithelial cells followed by strong innate immune system activation by dissemination of the proinflammatory colon luminal content, such as bacteria, into the sub-epithelial space (46, 47). Here, we hypothesized that Parp14 is involved in the anti-bacterial mucosal inflammation and set out to study the expression and function of Parp14 in a bacterial enteropathogen mouse model.

We used the mouse streptomycin-pretreatment model of *S*. Typhimurium colitis (8, 33). The streptomycin treatment transiently depletes the microbiota, and the subsequent *S*. Typhimurium colitis resembles many aspects of human disease, for example, epithelial erosion and massive infiltration of neutrophils (33). Our immunohistochemical analysis demonstrated that epithelial cells across the mucosal tissues in small intestine, cecum, and large intestine were Parp14-positive. Enterocytes, the absorptive cells primarily forming the intestinal barrier, were consistently Parp14-positive. To substantiate the immunohistochemical findings, we analyzed the published single-cell RNA-Seq data of eight different small intestine epithelial cell populations in the control and *S*. Typhimurium-infected mice (37). The expression of *Parp14* in the infected mice was pronounced in the enterocytes and Tuft cells. The latter ones are rare and functionally elusive cells found in the mucosal tissue with immunomodulatory potential (48). It is noteworthy that the mucosal tissues also contained some Parp14-positive cells, which were also positive for the F4/80 macrophage marker. This resembles what we and others previously reported for the large intestine in the oral DSS exposure mouse model of IBD and in IBD patients (35, 49). In the large intestine, it appeared that the most strongly Parp14-staining F4/80-positive cells were frequently embedded in the epithelial cell layer, having protrusions toward the gut lumen. Contacts with the luminal content might have driven the detected strong expression of Parp14, for example, with bacterial lipopolysaccharide (LPS), which is a known inducer of Parp14 expression in human and mouse macrophages *in vitro* (32, 35). Overall, Parp14 appeared to be expressed in macrophages and, in particular, in epithelial cells across the mucosal tissue of the mouse gastrointestinal tract.

We detected significant effects of Parp14 deficiency on the severity of salmonellosis. Out of the measured variables at day 1 and day 5 post-infection, the mouse weight, colon length, spleen weight, and liver weight were similar in the infected wt and Parp14-deficient mice. Also, the numbers of viable bacteria were mostly similar between the mouse genotypes in small intestine, large intestine, mesenteric lymph nodes, spleen, and liver. However, we witnessed increased numbers of viable bacteria in the liver of Parp14-deficient mice at day 1. At the same time point, we saw that the small intestine of Parp14-deficient mice had stronger epithelial erosion. We are tempted to speculate that the poor condition of the small intestine epithelium had contributed to the enhanced peripheral tissue invasion of *S*. typhimurium. In part, this effect could include defective Peyer’s patches and M cells therein, which are potent entry sites for peripheral tissue invasion of *S*. Typhimurium (50, 51). Interestingly, stronger epithelial cell erosion was not witnessed in the infected Parp14-deficient mice at day 5. Therefore, the deleterious effect of Parp14 deficiency in the small intestine appeared to be transient. At day 5, we witnessed lower amounts of viable bacteria in the large intestine of Parp14-deficient mice. At the same time, we saw that the large intestine of Parp14-deficient mice had stronger epithelial erosion, immune cell infiltration, and goblet cell loss. These days, five findings imply that the mucosal inflammation in the large intestine of infected Parp14-deficient mice was hyperactive, resolving the infection more efficiently, but, at the same time, it resulted in exacerbated tissue destruction. Overall, it appears that Parp14 is involved in the regulation of mucosal inflammation, or it influences the mucosal epithelial cell barrier integrity, or possibly both of these interconnected phenomena, in *S*. Typhimurium infection.

To possibly obtain mechanistic insights on the exacerbated salmonellosis in Parp14-deficient mice, we executed a bulk tissue RNA-Seq analysis of the large intestine supplemented with TaqMan qPCR assays. Systems-wide GO- and KEGG-based functional categorization of the RNA-Seq data led to identification of infection- and inflammation response-related transcriptomic signatures that were defective in the infected Parp14-deficient mice. The most notable KEGG hit was the IL-17 signaling pathway, which is a key regulatory pathway of mucosal inflammation triggered by the six-member IL-17 cytokine family (52). In this respect, it is noteworthy that CD4+ T cells of Parp14-deficient mice have difficulties *in vitro* to differentiate to Th17 cells (53, 54), the primary producers of IL-17A and IL-17F (52). Moreover, in a model of allergic airway inflammation, less IL-17-positive CD4+ T cells have been detected in the lungs and in the bronchoalveolar lavage samples of Parp14-deficient mice, in parallel with lower transcript and protein levels of IL-17A (54). However, our RNA-Seq analysis did not identify statistically strong, that is, *P*_adj_ < 0.05, evidence of lower *Il17* transcript levels in the infected Parp14-deficient mice. We detected downregulation of *Il17d* (Log2FC −0.48913; *P*-value 0.022107; *P*_adj_-value 0.255385) and *Il17f* (Log2FC −5.15979; *P*-value 0.011622; *P*_adj_-value 1). Yet, both RNA-Seq and TaqMan qPCR assays revealed lower transcript levels of genes classified in the KEGG hierarchy as IL-17 signaling pathway downstream effectors, or as IL-17 signaling pathway signature genes (55), such as *Il1b*, *Il6,* and *Ccl2*. This implies that the downstream effector step of the IL-17 signaling pathway was defective in the infected Parp14-deficient mice. However, the pleiotropic IL-6 cytokine is also an important upstream activator of the Th17 cell differentiation process (56). Furthermore, based on our TaqMan qPCR analysis and RNA-Seq with weak (*P*_adj_ > 0.05) statistical support (Log2FC −3.83621; *P*-value 0.039471; *P*_adj_-value 1), we detected compromised transcript levels of *Il23a*, encoding the p19 subunit of the heterodimeric p19/p40 IL-23 cytokine (57). IL-23 promotes the activation of effector functions of Th17 cells and is primarily produced by macrophages (57). Overall, it appears, based on our transcript-level functional approximations, that the infected Parp14-deficient mice were subject to a defective Th17 response possibly involving functionally compromised T cells and mononuclear phagocytes.

Parp14 was expressed by epithelial cells across the mucosal tissues in the mouse gastrointestinal tract. To experiment on the plausible epithelial cell functions of Parp14, we performed a comparative analysis of the published single-cell RNA-Seq data of small intestine epithelial cell types (37) with our bulk large intestine RNA-Seq data supplemented with TaqMan qPCR assays. This analysis led to the identification of three genes, that is, *ApoA1*, *Spink1,* and *Sst*, encoding apolipoprotein A1, serine protease inhibitor Kazal-type 1, and somatostatin, respectively. These genes, in particular *ApoA1*, were upregulated by *S*. Typhimurium infection in multiple small intestine epithelial cell subtypes (37). Yet, their expression was hampered in the large but not the small intestine of our *S*. Typhimurium-infected Parp14-deficient mice. In this respect, it is noteworthy, based on our histopathology analysis, that the large intestine suffered the most from Parp14 deficiency. ApoA1 is a component of the high-density lipoprotein particles, but it also has potent anti-inflammatory functions (58), for example, via neutralization of the effects of bacterial LPS (59–61). In respect to prior phenotypic work, an exacerbated DSS-induced colitis is known to develop in ApoA1-deficient mice (59), and ApoA1 mimetic peptide ameliorates the DSS-induced colitis (59). Other gastrointestinal murine disease models also support the anti-inflammatory functions of ApoA1 (60, 62). Serine protease inhibitor Kazal-type 1 (Spink1, formerly Spink3 in mouse) acts as the first line of defense against premature trypsinogen activation in the pancreas (63). However, it is also expressed elsewhere, including the gastrointestinal tract (64). To the best of our knowledge, Spink1 has not previously been implicated in the physiological response to a gastrointestinal bacterial infection. Somatostatin is a peptide hormone widely distributed in the central nervous system and endocrine organs, including the gastrointestinal tract (65). It has a plethora of functions in the gastrointestinal tract, including modulation of gastrointestinal motility as well as exocrine and endocrine secretion processes (65). Studies on its functions in enteric bacterial infections are scarce. Somatostatin inhibits TNF-α- or *Salmonella*-induced secretion of IL-8 and IL-1β by intestinal epithelial cells *in vitro* (66). The authors proposed that somatostatin could limit the inflammation induced in acute enteric diseases such as *Salmonella* gastroenteritis to prevent the development of chronic inflammation and the ensuing tissue damage (66). Taken together, our epithelial cell-centric analysis identified three genes having plausible protective functions in *Salmonella* infection and hampered expression in the *S*. Typhimurium-infected Parp14-deficient mice. Whether the detected defect was indeed due to the lack of epithelial cell-intrinsic functions of Parp14, or if it was caused by some extrinsic effect on epithelial cells, for example, the defective Th17 response, remains a subject of future studies.

In summary, our study with the systemic Parp14-deficient mice provides compelling *in vivo* evidence that Parp14 is an integral part of the physiological response to *S*. Typhimurium infection. The infected Parp14-deficient mice suffered from exacerbated histopathology. Using transcriptome-based functional approximations, we found evidence of a defective Th17 response and functionally hampered T cells and macrophages. We also found evidence of a defective epithelial cell-specific response involving genes (*ApoA1*, *Spink1*, *Sst*) with scarce prior knowledge of their functional significance in enteric bacterial infections. We conclude that Parp14 acts as a multi-cell-type pleiotropic regulator of mucosal inflammation. Further animal experimentation and *in vitro* work are needed to identify the exact phenotype-contributing cell type(s) spatially and temporally, for example, with cell lineage-specific Parp14 knockout mice in parallel with single-cell RNA-Seq studies, and to understand the functional contributions of the detected transcriptomic abnormalities in *S*. Typhimurium infection.

## Data Availability

The raw bulk mouse tissue RNA-Seq data has been deposited to NCBI (https://www.ncbi.nlm.nih.gov) Gene Expression Omnibus (GEO) database with accession number GSE284287. The published (37) mouse small intestine single -cell RNA-Seq data wasdata were analyzed and visualized at the Broad Institute Single Cell Portal (https://singlecell.broadinstitute.org/single_cell). All the other data that support the findings of this study are available in the Materials and Methods, Results, and/or Supplemental Material of this article.

## SUPPLEMENTAL MATERIAL

Data Set S1 spectrum.02971-25-s0001.xlsx Gene expression data.

Supplemental figures spectrum.02971-25-s0002.pdf Figures S1 to S6.

ASM does not own the copyrights to Supplemental Material that may be linked to, or accessed through, an article. The authors have granted ASM a non-exclusive, world-wide license to publish the Supplemental Material files. Please contact the corresponding author directly for reuse.
