# Supplementary material for: Exacerbated salmonellosis in poly(ADP-ribose) polymerase 14-deficient mice
Source: Microbiol Spectr. 2025 Dec 30;14(2):e02971-25. doi: 10.1128/spectrum.02971-25 (PMC12889102; doi:10.1128/spectrum.02971-25)
Supplement: Supplemental figures — Figures S1 to S6. [file spectrum.02971-25-s0002.pdf]

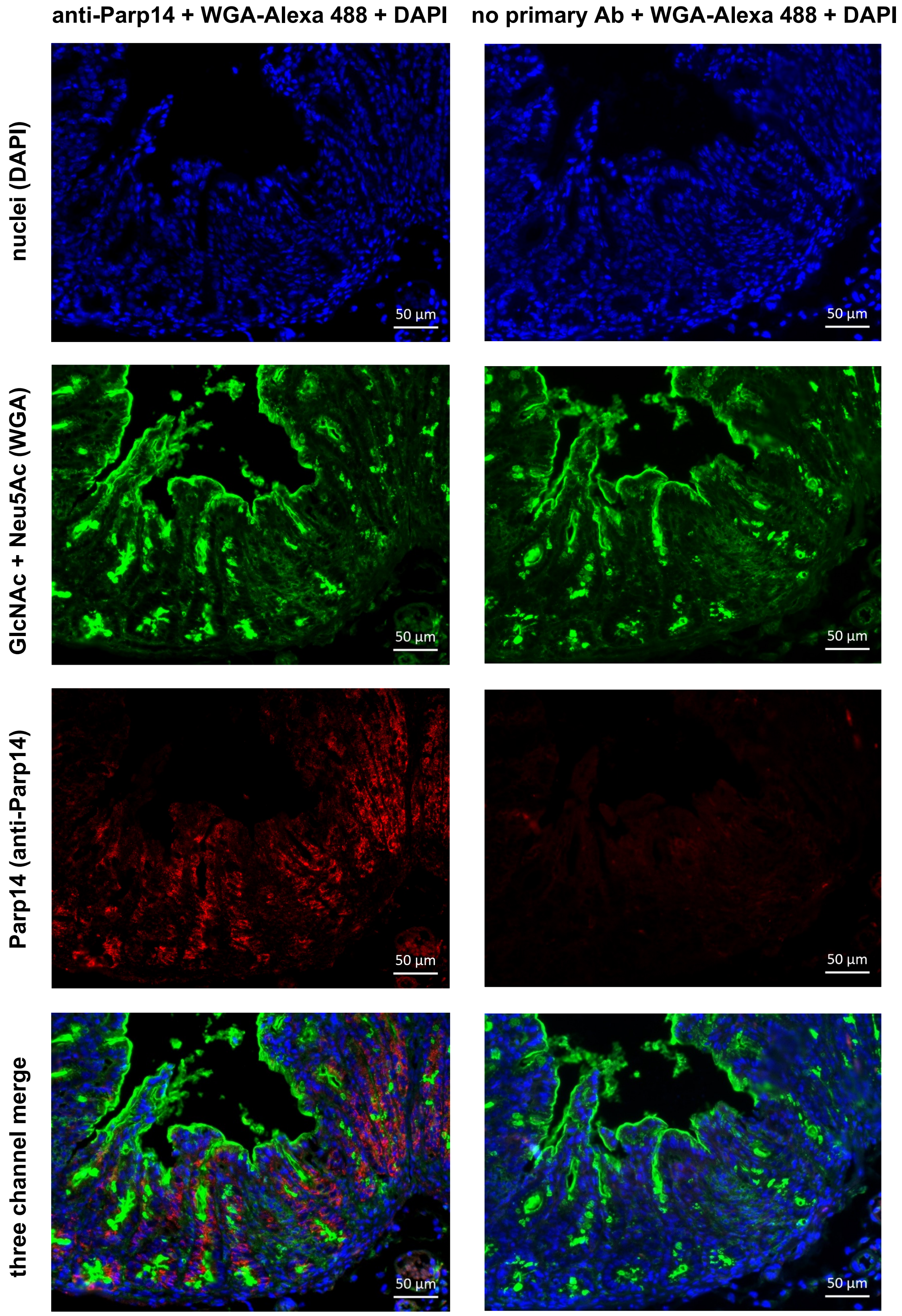

Figure S1. Vedantham et al.

Figure S1. Immunofluorescence staining of Parp14 in the mouse large intestine FFPE tissue sections. Tissue section from the *Salmonella* infected C57BL/6N mice (day 5) was analyzed for Parp14 (red channel, 1:500 dilution of anti-Parp14 antibody) as well as for N-acetylglucosamine (GlcNAc) and N-acetylneuraminic acid residues (Neu5Ac) [green channel, 5 µg/mL of wheat germ agglutinin(WGA)-Alexa 488 conjugate]. The N-acetylglucosamine and N-acetylneuraminic acid (sialic acid) residues are often found on cell membranes. Thereby, the WGA staining was used to visualize the overall cellular structure of the mucosal epithelium.

anti-Parp14 + anti-F4/80 + DAPI

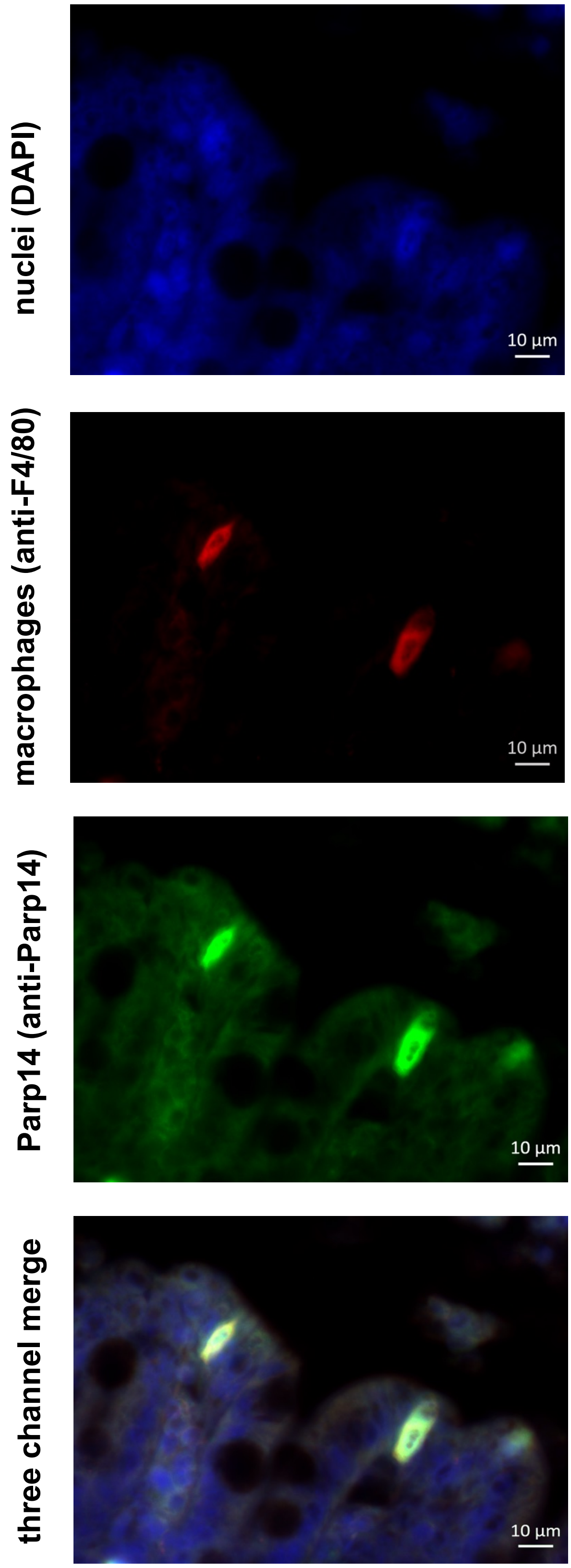

Figure S2. Vedantham et al.

Figure S2. Double immunofluorescence staining of Parp14 and the macrophage marker F4/80 in the mouse large intestine FFPE tissue sections. Tissue section derived from the *Salmonella* infected C57BL/6N mice (day 1) was analyzed for Parp14 (green channel, 1:500 dilution of anti-Parp14 antibody) and macrophage marker F4/80 (red channel). 63x objective image is shown (scale bar, 10 μm).

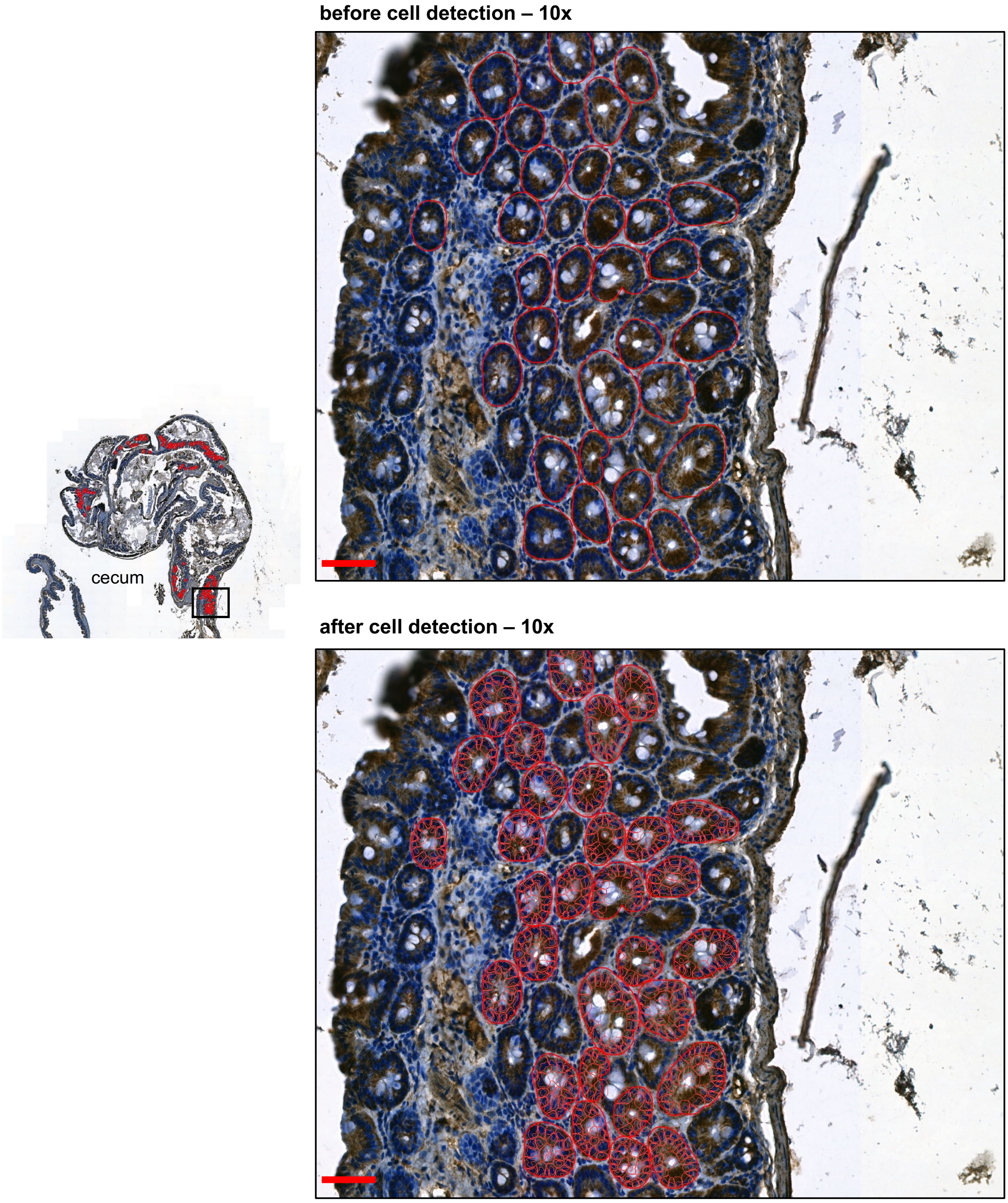

Figure S3. Vedantham et al.

Figure S3. QuPath-based quantitation of Parp14 staining in the mouse gastrointestinal tract FFPE tissue sections. Parp14 staining intensity was quantified with entire tissue sections by selecting 50-200 horizontal villus cross-sections (circled in red, before cell detection, scale bar, 50 µm) and thereby thousands of individual cells (mostly epithelial cells, circled in red after automatic cell detection, scale bar, 50 µm) per animal. The figure is an example of one cecum section Parp14 staining quantitation (see Fig. 3).

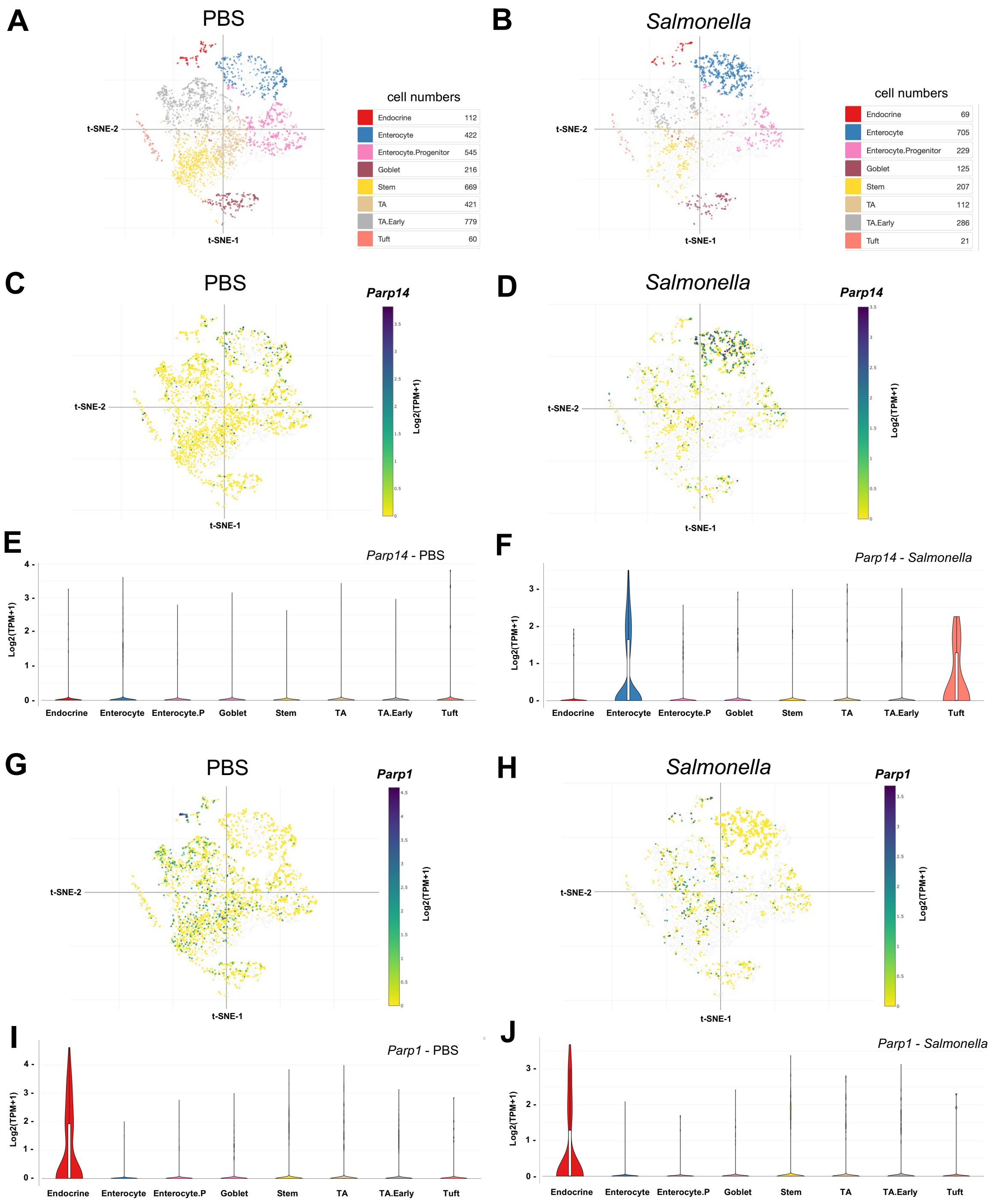

Figure S4. Vedantham et al.

Figure S4. Single cell RNA-Seq analysis of *parp14* expression in different mouse epithelial cell subtypes of the small intestine. A-B) Description of the identified mouse epithelial cell subtypes and their numbers in control vs. *Salmonella*-infected mice 2 days post-infection. The t-distributed stochastic neighbor embedding (t-SNE) method was used to visualize the data. **C-D)** Expression levels of *Parp14* in the different epithelial cell subtypes. The expression of *Parp14* is displayed in Log2(TPM+1)-values, that is, log2-transformed transcript per million-values. The t-distributed stochastic neighbor embedding (t-SNE) method was used to visualize the data. **E-F)** Distribution blot of *Parp14* expression levels in the different epithelial cell subtypes. **G-H)** Expression levels of *Parp1* in the different epithelial cell subtypes. The expression of *Parp1* is displayed in Log2(TPM+1)-values, that is, log2-transformed transcript per million-values. The t-distributed stochastic neighbor embedding (t-SNE) method was used to visualize the data. **I-J)** Distribution blot of *Parp1* expression levels in the different epithelial cell subtypes. All the data was analyzed and visualized using the single cell RNA-Seq data analysis and visualization interface at the Broad Institute Single Cell Portal (https://singlecell.broadinstitute.org/single_cell).

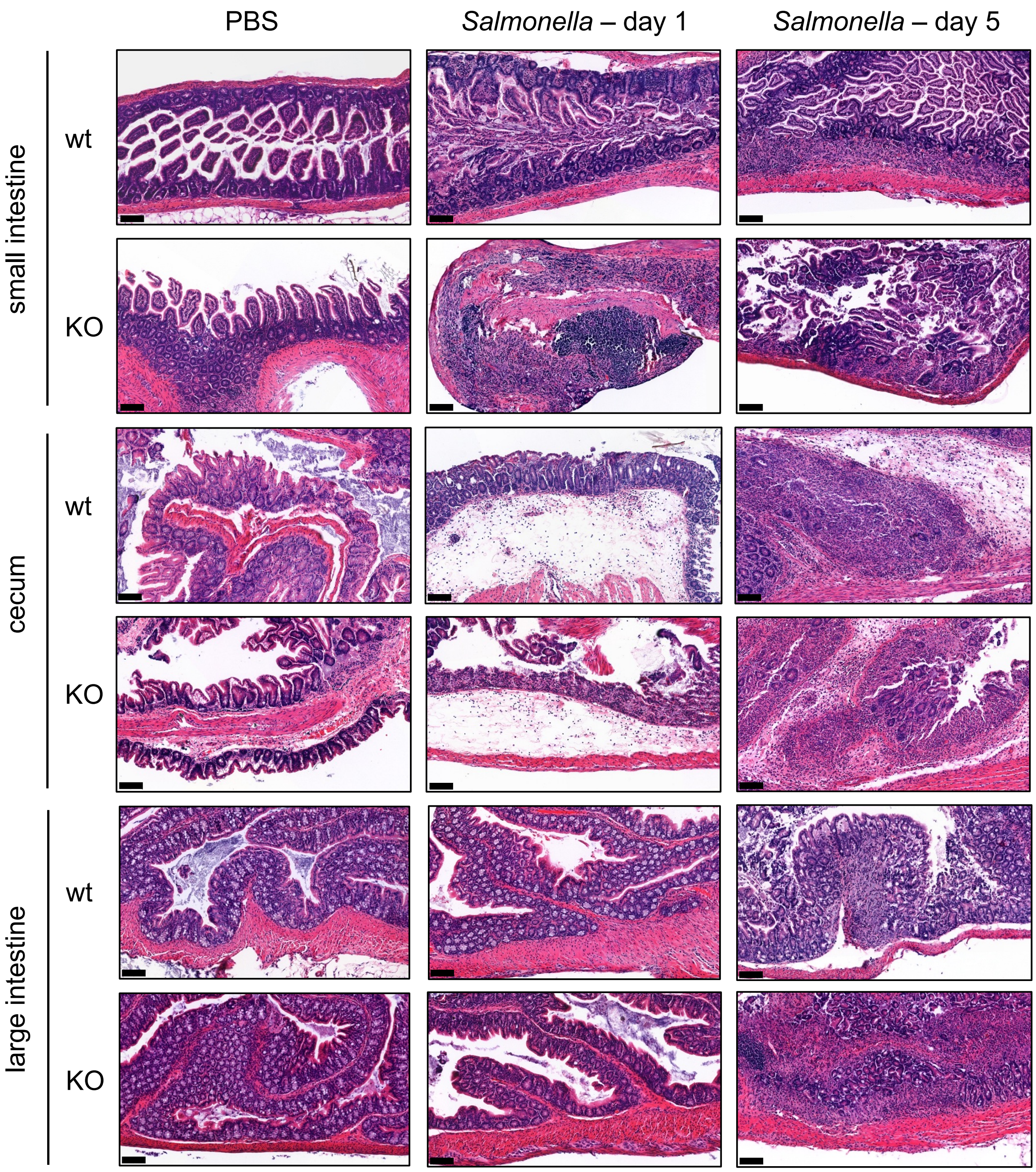

Figure S5. Vedantham et al.

Figure S5. Exacerbated gastrointestinal histopathology in *S.* Typhimurium infected Parp14 deficient mice. Representative H&E-stained tissue sections are shown to quantify histopathological variables, that is, epithelial erosion, tissue edema, immune cell infiltration and Goblet cell loss in distal small intestine, cecum, and large intestine (see Fig. 4A). The 10x and 30x air objective images are shown. The size of the scale bar in sub-panels refers to 100 μm (10x) and 50 μm (30x).

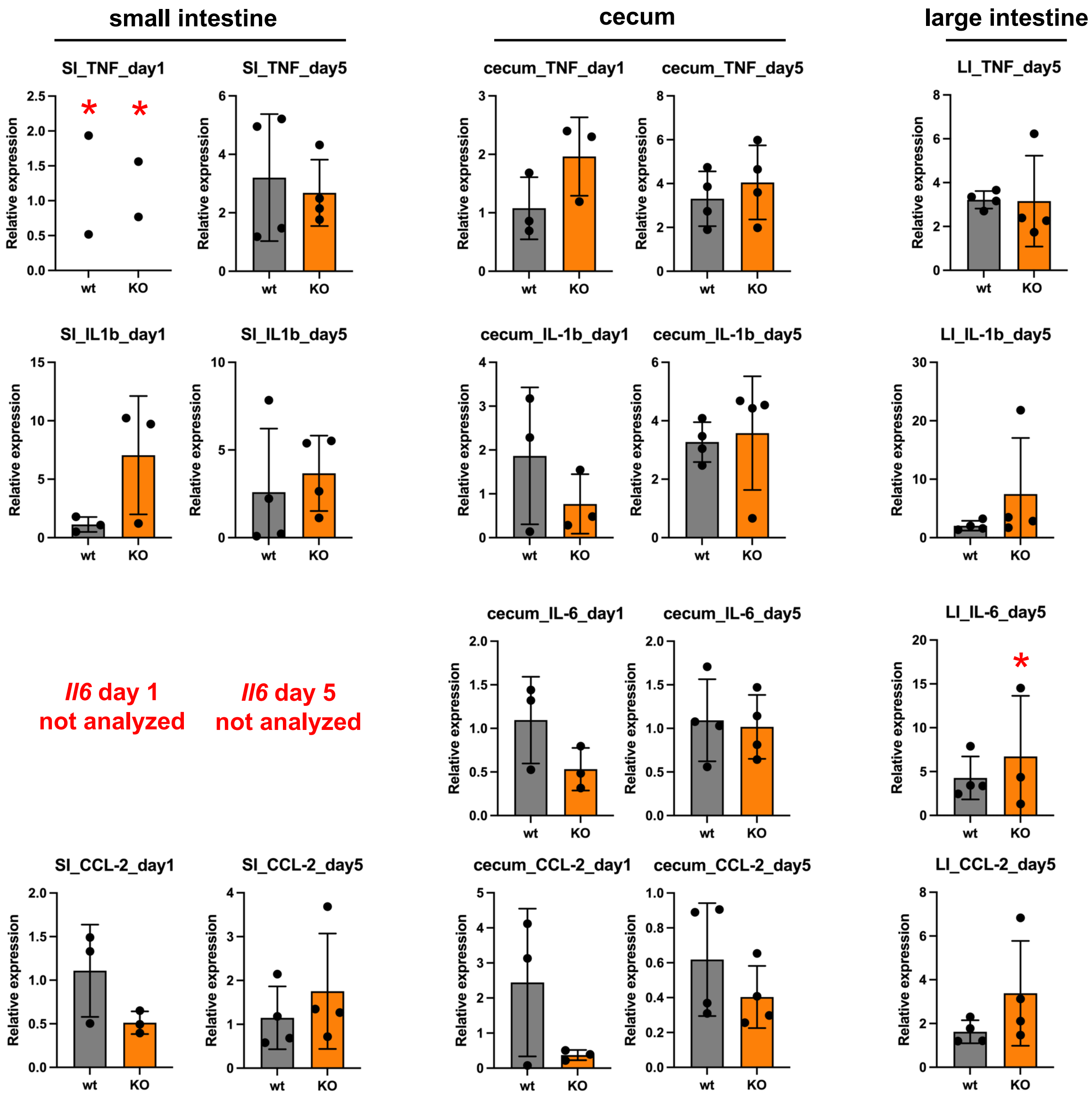

Figure S6. Vedantham et al.

Figure S6. TaqMan qPCR-based quantitation of cytokine expression in the small intestine, cecum and large intestine. The panels display the TaqMan qPCR data on relative gene expression with means and standard deviations. Samples were included in the displayed data analysis if they passed the 0.5 standard deviation Ct filter of replicate TaqMan runs. The red asterisks refer to experimental conditions where one or more samples did not pass this 0.5 Ct filter. The calibrators in all sub-panels are the mean dCq-values of the day 1 *Salmonella* infected wt mice. Statistical analyses were done with two-tailed unpaired t-test to compare experimental conditions with 3 or more biological replicates. No statistically significant differences between the wt and Parp14-deficient mice were detected.
